# Genome-Wide Analysis and Characterization of the *Aux/IAA* Family Genes Related to Floral Scent Formation in *Hedychium coronarium*

**DOI:** 10.3390/ijms20133235

**Published:** 2019-07-01

**Authors:** Yanguo Ke, Farhat Abbas, Yiwei Zhou, Rangcai Yu, Yuechong Yue, Xinyue Li, Yunyi Yu, Yanping Fan

**Affiliations:** 1 The Research Center for Ornamental Plants, College of Forestry and Landscape Architecture, South China Agricultural University, Guangzhou 510642, China; 2 College of Life Sciences, South China Agricultural University, Guangzhou 510642, China; 3 Guangdong Key Laboratory for Innovative Development and Utilization of Forest Plant Germplasm, South China Agricultural University, Guangzhou 510642, China

**Keywords:** *Hedychium coronarium*, *Aux/IAA*, floral scent, *HcIAA*

## Abstract

Auxin plays a key role in different plant growth and development processes, including flower opening and development. The perception and signaling of auxin depend on the cooperative action of various components, among which auxin/indole-3-acetic acid (Aux/IAA) proteins play an imperative role. In a recent study, the entire *Aux/IAA* gene family was identified and comprehensively analyzed in *Hedychium coronarium*, a scented species used as an ornamental plant for cut flowers. Phylogenetic analysis showed that the *Aux/IAA* gene family in *H. coronarium* is slightly contracted compared to *Arabidopsis*, with low levels of non-canonical proteins. Sequence analysis of promoters showed numerous *cis*-regulatory elements related to various phytohormones. *HcIAA* genes showed distinct expression patterns in different tissues and flower developmental stages, and some *HcIAA* genes showed significant responses to auxin and ethylene, indicating that *Aux/IAAs* may play an important role in linking hormone signaling pathways. Based on the expression profiles, *HcIAA2*, *HcIAA4*, *HcIAA6* and *HcIAA12*, were selected as candidate genes and *HcIAA2* and *HcIAA4* were screened for further characterization. Downregulation of *HcIAA2* and *HcIAA4* by virus-induced gene silencing in *H. coronarium* flowers modified the total volatile compound content, suggesting that *HcIAA2* and *HcIAA4* play important roles in *H. coronarium* floral scent formation. The results presented here will provide insights into the putative roles of *HcIAA* genes and will assist the elucidation of their precise roles during floral scent formation.

## 1. Introduction

Auxin plays a substantial role in several aspects of plant growth and development, like cell division, apical dominance, vascular differentiation, lateral/adventitious root formation and fruit and flower development [1,2,3]. *Aux/IAA* genes constitute one of the three major classes of primary auxin-responsive genes, including *SAUR* (Small Auxin UP RNA) and *GH3* (Gretchen Hagen 3) [4,5]. Aux/IAA proteins can act as transcriptional repressors through interactions with ARF (auxin response factor) proteins. Aux/IAAs inactivate ARFs, which can be either transcriptional repressors or activators of primary auxin-responsive genes [6,7]. *Aux/IAAs* are the primary responsive auxin genes, most of which are short-lived in the cytosol and nucleus [1,8,9]. Aux/IAA proteins are normally conserved with four domains known as domain I to domain IV, although proteins missing one or two domains were also included in this gene family [1]. Domain I consists of a leucine-rich repeat motif symbolized by “LxLxL” and acts as an active repression domain that can interact with the corepressor protein TOPLESS (TPL) [10,11]. Domain II is highly conserved with the degron sequence (GWPPV), leading towards Aux/IAA protein instability by ubiquitin degradation) [12,13,14,15]. Domain III and IV at the C-terminus intercede homo- and heterodimerization among Aux/IAA proteins and/or auxin ARF proteins [1,16,17]. Usually, auxin-responsive *cis*-elements (AuxREs) are present at the promoter regions of auxin-responsive genes that bind to ARFs to regulate the expression of auxin-mediated genes. To regulate auxin-responsive genes, Aux/IAA proteins do not bind directly to AuxREs; however, they interact with ARFs by controlling ARF activity [18]. The study of auxin regulation and activity is highly complex because of the extensive number of *Aux/IAA* and *A*RF family members, expression patterns, variations, and auxin-mediated transcriptional and post-transcriptional regulations.

Formerly, a large number of *Aux/IAA* genes were identified and characterized by mutant analysis, especially in *Arabidopsis* and tomato. In *Arabidopsis*, a functional mutation in *IAA8* altered lateral branches, curled leaves, shortened primary inflorescence stems, decreased shoot apical dominance, induced the formation of abnormal flower organs (bent stigmas, short petal and stamen) and reduced the jasmonic acid level in the flowers. In tomato, compared with wild-type plants, *SlIAA15* suppressed transgenic lines by showing a higher number of xylem cells. The monoterpene content, including β-phellandrene, α-terpinene, γ-element, α-humulene and, β-caryophyllene, in trichome exudates was reduced significantly in *Sl-IAA15* downregulated leaves [19]. However, the silencing of the *Sl-IAA27* gene showed multiple phenotypes related to vegetative and reproductive growth [20]. Furthermore, *Sl-IAA27* silencing resulted in the downregulation of strigolactone biosynthesis by regulating the genes involved in strigolactone synthesis [21]. Overall, *Aux/IAA* plays a key role in monocots and dicots plants by affecting the development of flowers, roots and stems. It also affects some secondary metabolism, such as the biosynthesis of volatile compounds [22,23,24]. *Aux/IAA* gene family members have been identified in numerous plant species, including *Arabidopsis* [25], rice [3], maize [26], tomato [27], *Vitis vinifera* [28], *Eucalyptus* [29] and papaya [30]. However, the function of *Aux/IAA* family members in *Hedychium coronarium* is still unknown.

*H. coronarium* is a perennial herb frequently cultivated as a cut flower or garden plant in tropical and subtropical regions. The flower is famous for its fragrance and medicinal importance [31]. The blooming of flowers results in the emission of a blend of volatile compounds mainly consisting of monoterpenes (linalool, 1,8-cineole, (*E*)/(*Z*)-β-ocimene), sesquiterpenes (β-caryophyllene, α-farnesene) and some benzenoids [32,33,34,35]. Similar to color and shape, floral fragrance is also an important characteristic of any ornamental plant, refining its esthetic and economic value [36]. To the best of our knowledge, a genome-wide analysis of the *Aux/IAA* gene family in *H. coronarium* has not been performed, and the function of *Aux/IAA* genes in floral scent formation is still unknown. In the current study, we identified *Aux/IAA* family genes in *H. coronarium* genomes and analyzed their sequence characteristics, genomic structures, phylogeny and *cis*-regulatory elements. The spatiotemporal differential expression patterns of *Aux/IAA* in different tissues/organs and at different flower developmental stages were also studied. Additionally, we evaluated the roles of *Aux/IAA* members in floral scent formation through their response to various hormonal treatments. Moreover, we identified two nuclear-localized *Aux/IAA* genes (*HcIAA2* and *HcIAA4*) that are involved in floral scent formation, as demonstrated by virus-induced gene silencing. Our findings will provide novel insights into the functions of *Aux/IAA* and will assist scientists in future studies on elucidating the precise biological functions of *Aux/IAA* genes in *H. coronarium*.

## 2. Results

### 2.1. Identification and Sequence Analysis of the Aux/IAA Gene Family in H. coronarium

According to a pBLAST search, a total of 35 candidate gene models were originally found. The annotation of these gene models was assessed by using *H. coronarium* transcriptome data. Falsely predicted *Aux/IAA* gene models were curated manually. A total of 27 *H. coronarium Aux/IAA* genes were identified and named *HcIAA1*–*HcIAA27*. Detailed information on these *HcIAA* genes, including gene names, sequence IDs, exon number, genome location, open reading frame (ORF) lengths, protein molecular weight (MW), length of the protein sequence and isoelectric point (*pI*), is listed in Table 1.

The predicted HcIAA proteins vary in size from 155 (HcIAA7) to 352 amino acids (HcIAA24) with molecular masses ranging from 16 to 39 kDa (Table 1). The theoretical isoelectric points also differ greatly from 4.66 (HcIAA17) to 10.15 (HcIAA23), showing that they may have roles in diverse microenvironments. Pairwise analysis of HcIAA protein sequences revealed that the identity differs widely from 86.9% (between HcIAA9 and HcIAA10) to 14.4% (between HcIAA24 and HcIAA27) (Table S2). A similar large variation was reported in *Arabidopsis* [17], tomato [37] and *Eucalyptus* [29].

### 2.2. Multiple Sequence Alignment and Phylogenetic Analysis of HcIAA Genes

**Figure 1:**
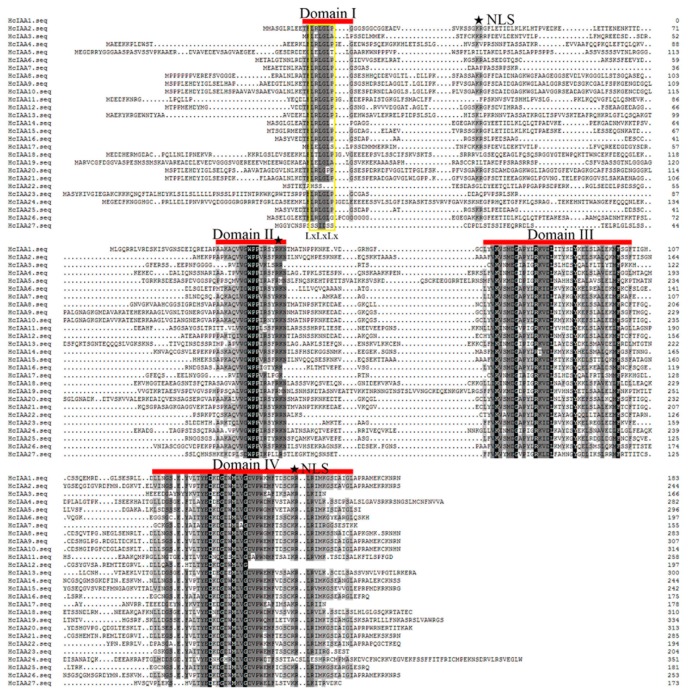
Multiple sequence alignment of the full-length HcIAA proteins obtained with Clustal W and manual correction. Domains I–IV of the *H. coronarium* IAA proteins are indicated with red lines. Color shading indicates identical and conserved amino acid residues. Nuclear localization signals (NLSs) are indicated by filled stars. The amino acid position is given to the right of each sequence. The yellow color box showed a typical LxLxLx motif present in domain I.

Alignment of the amino acid sequences of *H. coronarium* Aux/IAAs revealed that the typical four highly conserved domains (domains I, II, III and IV) were present in the majority of HcIAA proteins. A typical LxLxLx motif was present in domain I of the majority of HcIAA proteins, except HcIAA1 and HcIAA22. The consensus sequence (T/LELRLGLPG) in domain I was not well conserved in HcIAA3, HcIAA5 and HcIAA17 (Figure 1), and the conserved degron sequence VGWPP in domain II, which is important for degradation, was not found in HcIAA27. HcIAA12 was the only member that contained a truncated domain IV. In most of *H. coronarium* Aux/IAA proteins, two kinds of putative nuclear localization signals (NLS) were detected. The first NLS has a bipartite structure encompassing a conserved basic doublet, KR, between domains I and II and the next NLS is a basic residue-rich region situated in domain IV (Figure 1). Majority of the HcIAA proteins consist of both types of NLS and are hence most likely localized to the nucleus, consistent with their transcriptional activity. However, HcIAA12 and HcIAA24 lack the SV40-type NLS, whilst HcIAA11, HcIAA18, HcIAA19 and HcIAA27 lack the bipartite NLS. These putative NLSs suggest that HcIAAs are nuclear-located proteins (Figure 1).

The Aux/IAA family in *H. coronarium*, with 27 members, is slightly contracted compared with the 31 in *Oryza sativa*, 31 in *Zea mays* and 29 members in *Arabidopsis*. Its size resembles to that of tomato and *Eucalyptus*, both of which contain 26 members [29,37].

**Figure 2:**
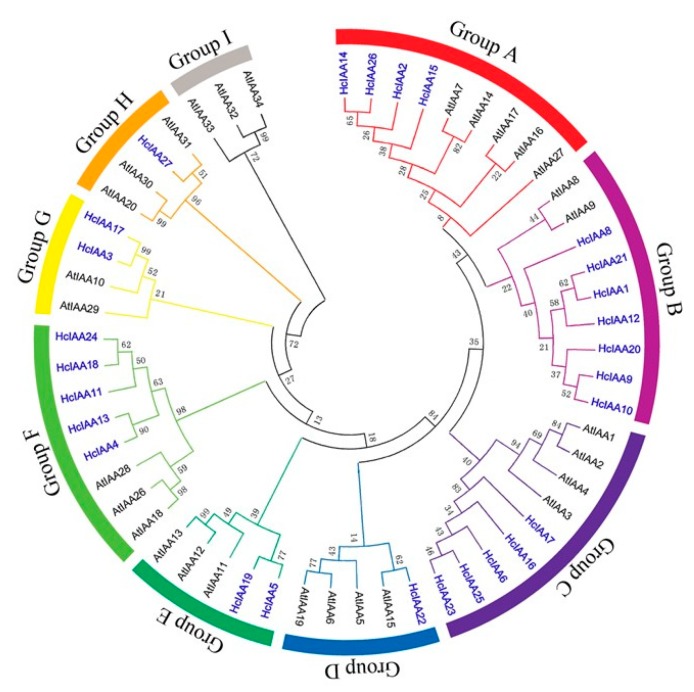
Phylogenetic analysis of *H. coronarium* and Arabidopsis Aux/IAA proteins. Full-length protein sequences were aligned by using the Clustal X 2.1 program. The phylogenetic tree was constructed by using MEGA 6 software and the neighbor-joining method with predicted Aux/IAA proteins. Bootstrap values are indicated at each node. Each Aux/IAA group (A–I) is indicated by a specific color. HcIAAs are noted in blue and bold.

To evaluate the relationship between *H. coronarium* and Arabidopsis Aux/IAAs, phylogenetic analysis was carried out by using the predicted full-length amino acid sequences of Aux/IAAs from *H. coronarium* and *Arabidopsis*. All Aux/IAA proteins were categorized into nine distinct groups named A-I (Figure 2). With reference to *Arabidopsis*, two groups (D and H) are contracted, and two groups (B and F) are expanded in *H. coronarium*. Group B contains seven genes in *H. coronarium* but only three members in *Arabidopsis*, while group F consists of five genes in *H. coronarium* and contains three members in *Arabidopsis*. The non-canonical group H, which lacks the conserved domain II, contains three members (AtIAA20, AtIAA30 and AtIAA31) in *Arabidopsis* but only one member in *H. coronarium* (HcIAA27; Figure 2). Group I, which also gathers non-canonical Aux/IAAs of *Arabidopsis*, is absent in *H. coronarium*. Overall, the non-canonical Aux/IAAs are overrepresented in *Arabidopsis* with six genes (AtIAA20, AtIAA30, AtIAA31, AtIAA32, AtIAA33 and AtIAA34), whereas only one was found in *H. coronarium* (HcIAA27).

### 2.3. Gene Structure and Motif Composition of HcIAA Genes

**Figure 3:**
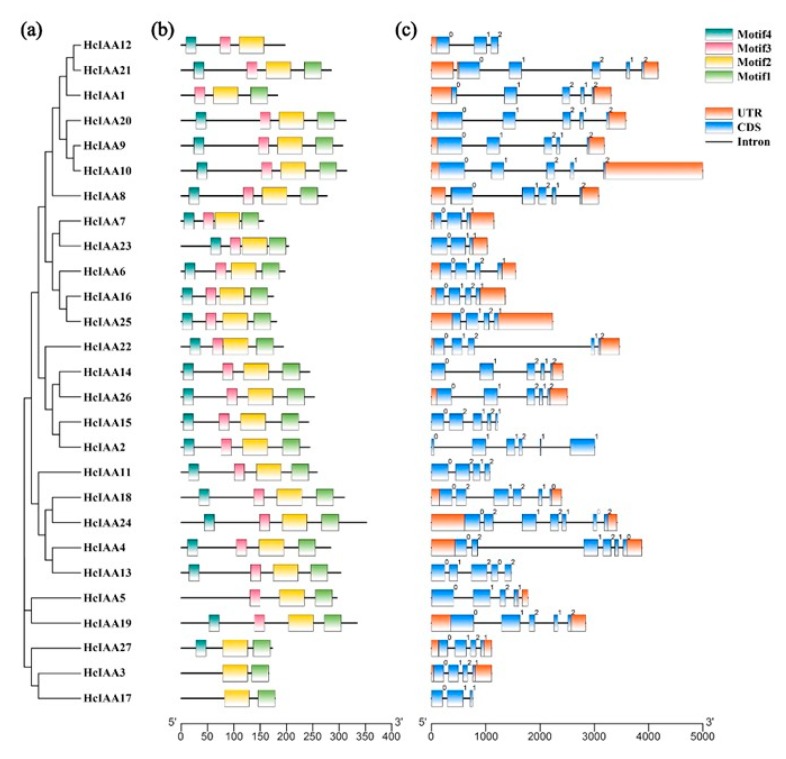
Phylogenetic relationships, motif distribution analysis and gene structure in *Aux/IAA* genes from *H. coronarium*. (**a**) The phylogenetic tree was constructed based on the full-length sequences of *H. coronarium* Aux/IAA proteins using MEGA 6 software. (**b**) The motif distribution in *H. coronarium* IAA proteins. By using a MEME web server, motifs of Aux/IAA proteins were analyzed. Four motifs representing domains I, II, III and IV are mapped on all of the Aux/IAA proteins in different colors. (**c**) Exon–intron structure of *H. coronarium Aux/IAA* genes. Orange boxes indicate untranslated 5′- and 3′-regions; blue boxes indicate exons; black lines indicate introns. The number indicates the phases of corresponding introns.

Schematic structures of *HcIAA* genes are shown in Figure 3. The MEME web server was used to analyze the domain distributions of HcIAA proteins. Four different conserved domains were mapped (Figure 3b). Most of the HcIAA proteins contain the four typical domains, while some of the IAA proteins have truncated domains, such as motif 4 (domain I), which is missing in HcIAA1, HcIAA3, HcIAA5 and HcIAA17; motif 3 (domain II), which is missing in HcIAA27, HcIAA3 and HcIAA17; and motif 1 (domain IV), which is missing in HcIAA12 (Figure 3b). The number of introns in all *HcIAA* genes was between two and six. The coding sequences of most (69%) of the *HcIAA* genes are disrupted by three or four introns, and the intron positions and phases are well conserved (Figure 3c). Variations were observed in some members involving mainly the loss of one intron (*HcIAA7*, *HcIAA12*, *HcIAA17* and *HcIAA23*) and, in some cases, the gain of one or more additional intron (*HcIAA2*, *HcIAA4*, *HcIAA18* and *HcIAA24*). The exon–intron organizations of all identified *HcIAA* genes were analyzed to gain more insight into the evolution of the Aux/IAA family in *H. coronarium*. Further analyses indicated that the distribution of introns and the intron phase coincided with the phylogenetic alignment of *HcIAA* genes (Figure 3a).

### 2.4. Analysis of Hormone-Related cis-Elements in the Promoter Regions of HcIAA Genes

**Figure 4:**
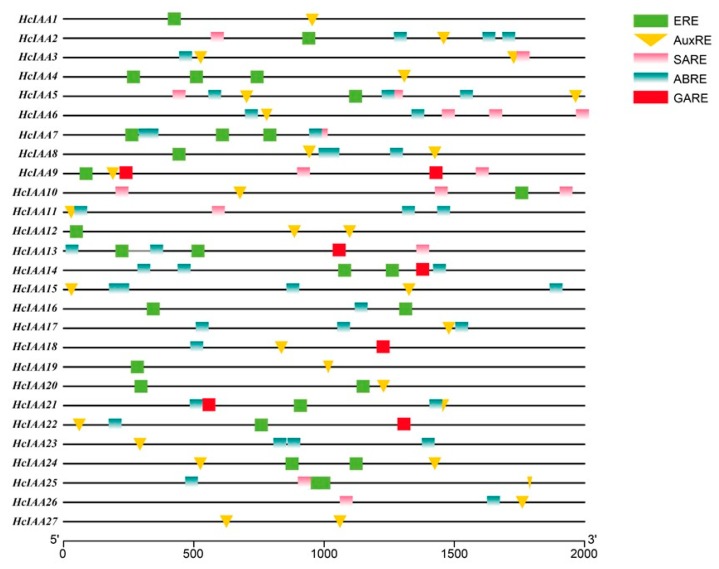
Analysis of specific *cis*-elements in promoters. The 2000 bp promoter sequences of *HcIAA* genes were used to analyze specific hormone-related *cis*-elements, including AuxRE, SARE, GARE, ERE and ABRE, which are color-coded. The AuxRE element is indicated with an inverted triangle.

The 2000 bp upstream promoter regions were scanned to identify hormone-related *cis*-elements to gain insight into how the expression levels of *HcIAA* genes responded to hormonal stimuli. The results revealed that the majority (23 out of 27) of the *HcIAA* promoters contained AuxREs as either a degenerate (TGTCCC) or conserved (TGTCTC) motif. Interestingly, 18 out of the 27 *HcIAA* promoters contained conserved ethylene-response motifs (AWTTCAAA). Additionally, several kinds of hormone-related *cis*-elements were present in the promoter regions of *HcIAA* genes, such as *HcIAA2*, *HcIAA9* and *HcIAA21*. One AuxRE, one SARE, one ERE and three ABREs were present in the promoter of *HcIAA2*; one AuxRE, two GAREs, two SAREs and one ERE were present in the promoter of *HcIAA9* and one AuxRE, two ABREs, one ERE and one GARE were present in the promoter of *HcIAA21* (Figure 4). Moreover, more than three ABREs were found in the promoters of *HcIAA5* and *HcIAA15*. The existence of these *cis*-regulatory elements shows a potential regulation of the *Aux/IAA* genes not only by auxin but also by other hormones.

### 2.5. Expression Profiling of HcIAA Genes in Different Organs/Tissues

**Figure 5:**
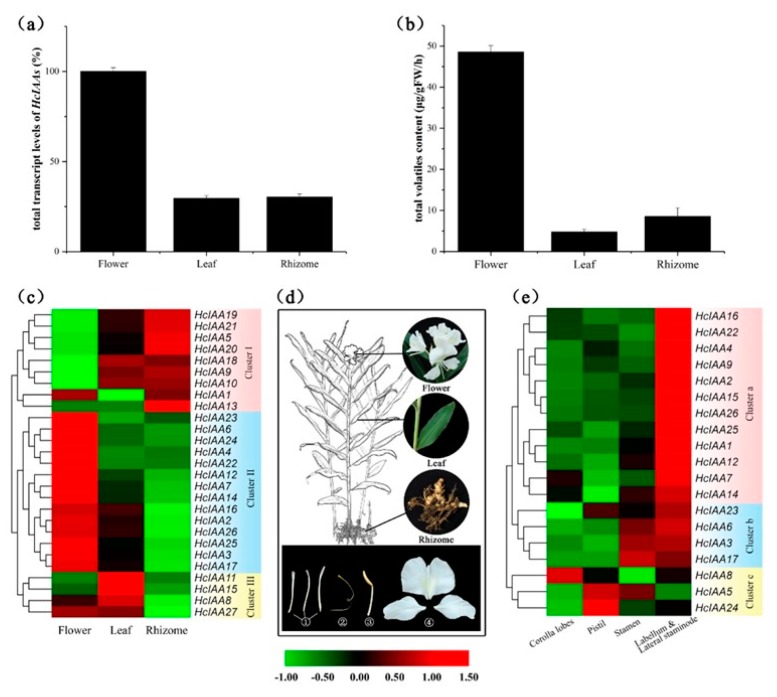
Expression profiles of *HcIAA* genes in various organs and tissues. (**a**) Total transcript levels of *HcIAAs* in flower, leaf and rhizome. (**b**) The total volatile contents in flower, leaf and rhizome. (**c**) Changes in the expression levels in three organs (flower, leaf and rhizome), which are schematically depicted above the displayed qRT-PCR data, are relative to RNA accumulation levels. Levels of downregulated expression (green) and upregulated expression (red) are shown on a log^2^ scale from the highest to the lowest expression for each *HcIAA* gene. (**d**) Pictorial view of different organs/tissues ((1) corolla lobes, (2) pistil, (3) stamen, (4) labellum and lateral staminode) of *H. coronarium.* (**e**) Changes in the expression levels in different parts of the flowers for 19 *HcIAA* genes, which are schematically depicted above the displayed qRT-PCR data, are relative to RNA accumulation levels. Levels of downregulated expression (green) or upregulated expression (red) are shown on a log^2^ scale from the highest to the lowest expression for each *HcIAA* gene.

Total volatile contents and total transcript levels of the 27 *Aux/IAAs* were checked from three organs, including the flower, leaf and rhizome. In full-boom *H. coronarium* flowering plants, the mRNA accumulation of total *HcIAAs* reached its highest level, while low-level expression was observed in the leaves and rhizomes (Figure 5a). Similarly, the maximum amounts of volatiles were found in the flowers, while low amounts were detected in the leaves and rhizomes (Figure 5b). To gain insight into the spatial expression patterns of *HcIAA* genes, transcript levels were assessed by qRT-PCR in different plant tissues and organs. The relative transcript accumulation of all the *HcIAA* genes is presented in a heat map, and hierarchical clustering allowed us to group all the expression patterns into distinct clusters. Three clusters are shown in the heat map for different organ expression levels (Figure 5c). Many members of the three clusters were expressed in flowers, except *HcIAA9*, *HcIAA10*, *HcIAA19, HcIAA20* and *HcIAA21*, which belong to cluster I. However, cluster I was preferentially expressed in vegetative organs (leaf and rhizome). All members in cluster II had higher expression levels in the flowers than in the vegetative organs. Members of cluster III were highly expressed in the leaves, but they diverged in their differential expressions in the rhizome when compared with cluster I. Notably, *HcIAA13* was the only gene that was specifically expressed highly in the rhizome (Figure 5c). Interestingly, *HcIAA4*, *HcIAA6*, *HcIAA22, HcIAA23* and *HcIAA24* showed the highest specific expression in flowers. Furthermore, the flower was divided into four different parts, including mixed labellum and lateral staminode, corolla lobes, pistil and stamen (Figure 5d), and *HcIAA* genes that were expressed in flowers were selected for further analysis in detailed tissues (Figure 5e). A vast majority of members belonging to cluster a and cluster b showed the highest expression level in scented tissues (mixed labellum and lateral staminode), except *HcIAA5*, *HcIAA8* and *HcIAA24*, which belong to cluster c. The members of cluster a were preferentially expressed in mixed labellum and lateral staminode compared to cluster b (Figure 5e).

### 2.6. Expression of HcIAA Genes at Different Flower Developmental Stages

**Figure 6:**
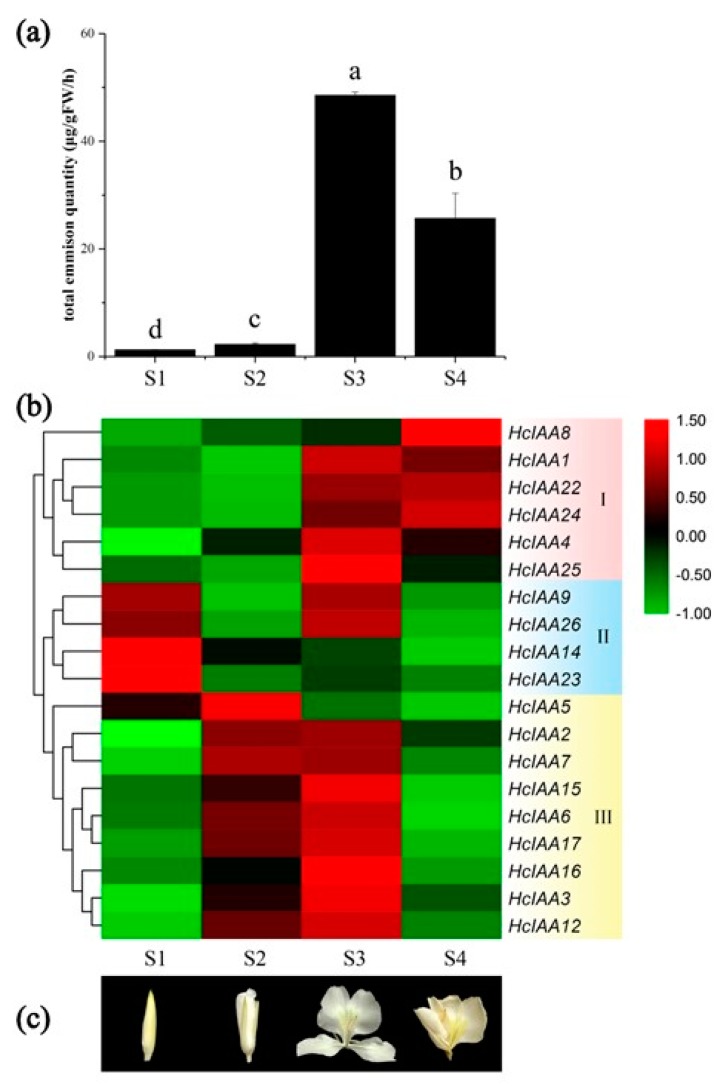
Heat map of the expression of *HcIAA* genes during different flower developmental stages. (**a**) The amount of floral volatiles emitted during developmental stages (S1, S2, S3 and S4). (**b**) The hierarchically clustered heat map was constructed with the relative expression qRT-PCR data for 19 *HcIAA* genes (indicated on the right) in four development stages (shown at the down). Levels of downregulated expression (green) or upregulated expression (red) are shown on a log^2^ scale from the highest to the lowest expression for each *HcIAA* gene. (**c**) Different flower developmental stages are shown as pictures.

The formation of floral volatile compounds is closely associated with developmental processes in *H. coronarium* [29]. The total emission of flower volatile compounds was sampled by headspace collection and analyzed by gas chromatography–mass spectrometry at four stages (Figure 6a). The amount of emitted volatile compounds was low at the bud period (S1) and increased slightly at the initial flowering stage (S2), while the emissions continuously increased during the opening stage, reaching the highest level at the blooming period (S3) and declining thereafter at senescence (S4) (Figure 6a). To elucidate the functions of the *HcIAA* genes during the flower developmental period, their expression levels at four different developmental stages were categorized into three groups by heat map software. Several *HcIAA* genes (*HcIAA1*, *HcIAA8*, *HcIAA22* and *HcIAA24*), which belong to Group I, showed a continuous increase in the expression level during the developmental process. However, some *HcIAA* genes, including *HcIAA9*, *HcIAA14*, *HcIAA23* and *HcIAA26*, which belong to Group II, were gradually reduced (Figure 6b). Within Group I, *HcIAA4* and *HcIAA25* dramatically peaked at stage S3 and then declined significantly at stage S4. Members of both Groups I and III were most highly expressed at the full-bloom stage (S3), but Group I showed a dramatic increase from S2 to S3, whereas those of Group III maintained a high expression level from S2. The expression levels of most *HcIAA* genes were significantly changed during flower development, indicating their potential function during the process of flower development and floral scent emission.

### 2.7. Expression of HcIAA Genes in Response to Hormone Treatments

**Figure 7:**
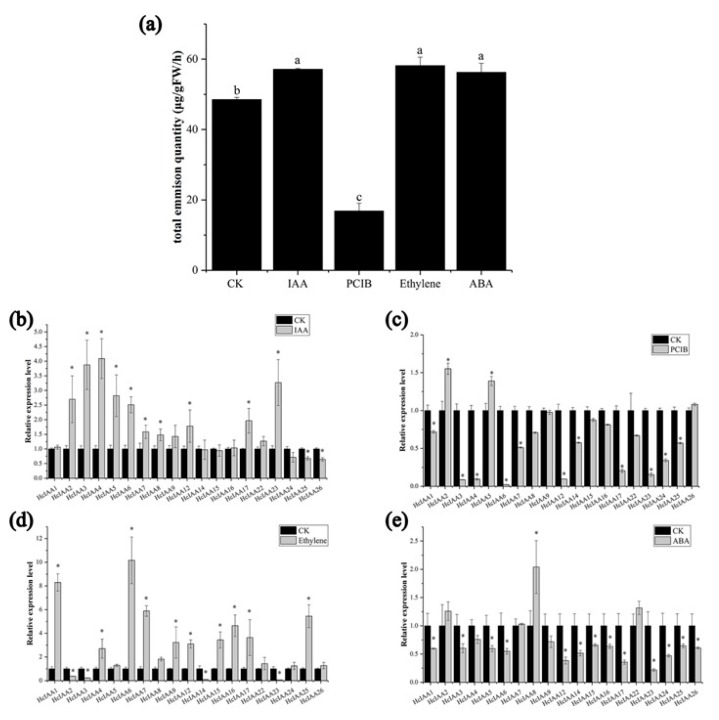
Relative expression levels of *HcIAA* genes and emission of floral volatiles under different hormonal treatments. (**a**) The amount of floral volatiles during different treatment. The expression of *HcIAA* genes in response to (**b**) IAA, (**c**) PCIB, (**d**) ethylene and (**e**) ABA treatments was analyzed by qRT-PCR. The expression levels of *HcIAA* genes in control flowers were set to a value of 1. The expression levels of *HcIAA* genes in IAA (100 μM), PCIB (1.5 mM), ethylene (10 μL/L) and ABA (200 μM) treated flowers were compared to a mock treatment for relative mRNA levels. Error bars represent standard deviations from three biological replicates. Significant differences (* *p* < 0.05) between the hormone-treated samples and control are indicated by an asterisk.

Auxin, ABA and ethylene are three major hormones involved in flower development and senescence [38,39]. PCIB, as an auxin signal inhibitor, also plays key roles in auxin signal transduction [40,41,42,43]. The total percentage of volatile compounds of *H. coronarium* flowers increased 16%, 20% and 21% under ABA, ethylene and IAA treatment, respectively, but decreased 52% with PCIB treatment (Figure 7a). The expression levels of *HcIAA* genes were verified by qRT-PCR under IAA, PCIB, ethylene and ABA treatments. The expression levels of *HcIAA3*, *HcIAA4* and *HcIAA23* were significantly upregulated by three-fold when treated with auxin, while *HcIAA25* and *HcIAA26* were reduced (Figure 7b). Under PCIB treatment, *HcIAA3*, *HcIAA4*, *HcIAA6* and *HcIAA12* were significantly reduced, while *HcIAA2* and *HcIAA5* were slightly upregulated (Figure 7c). Under ethylene treatment, *HcIAA1*, *HcIAA6*, *HcIAA7*, *HcIAA16* and *HcIAA25* were significantly upregulated, while *HcIAA14* and *HcIAA23* were downregulated (Figure 7d). The expression level of *HcIAA8* was upregulated under ABA treatment, whereas most genes were downregulated (Figure 7e).

### 2.8. Subcellular Localization of HcIAA Candidate Genes

**Figure 8:**
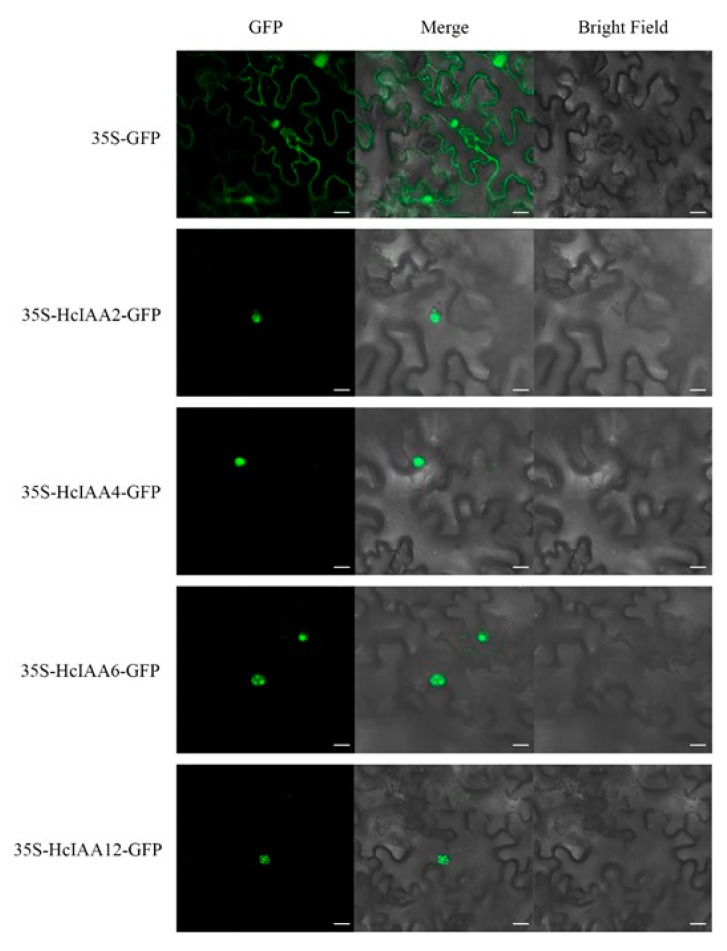
Subcellular localization of HcIAA2, HcIAA4, HcIAA6 and HcIAA12 proteins. HcIAA2-GFP, HcIAA4-GFP, HcIAA6-GFP and HcIAA12-GFP fusion proteins were transiently expressed in *N. benthamiana* leaves, and their subcellular localization was examined by confocal laser scanning microscopy. The merged pictures of the green fluorescence channel (left panels) and the corresponding bright field (right panels) are shown (middle panels). The scale bar indicates 20 µm.

To identify the best candidate gene(s) potentially involved in floral scent formation, we defined several criteria for further functional characterization in plants: Transcript abundance in flower organs; high expression in the labellum and lateral staminode, which are the main parts that emit floral scent; responsiveness to IAA and PCIB, which significantly influence flower scent formation; and expression patterns during flower developmental stages that match the emission of volatile compounds. The combination of all these criteria is presented as a Venn diagram (Supplement Figure S1). *HcIAA2*, *HcIAA4*, *HcIAA6* and *HcIAA12* were identified as the most suitable candidates that matched the criteria. The results of the prediction suggested that the candidates were localized in the nucleus (Figure 1). To experimentally verify subcellular localization, the full-length sequences of *HcIAA2*, *HcIAA4*, *HcIAA6* and *HcIAA12* were fused to a GFP reporter gene and transferred to *N. benthamiana* leaves, which were subsequently analyzed for transient GFP expression by confocal laser scanning microscopy. The results revealed that the green fluorescence of HcIAA2-GFP, HcIAA4-GFP, HcIAA6-GFP and HcIAA12-GFP was all located in the nucleus (Figure 8), which clearly indicated that the candidate HcIAA proteins are targeted to the nucleus. Similarly, numerous Aux/IAA proteins from different plant species have been identified, which are targeted to the nucleus, from which EgrIAA4 is among one of them, which is located exclusively in the nucleus [44,45]. Thus, HcIAA2, HcIAA4, HcIAA6 and HcIAA12 proteins were located in the nucleus as predicted and were able to mediate an auxin response in vivo consistent with their transcriptional activity.

### 2.9. Silencing of HcIAA2 and HcIAA4 Altered the Flower Volatile Compound Amount

**Figure 9:**
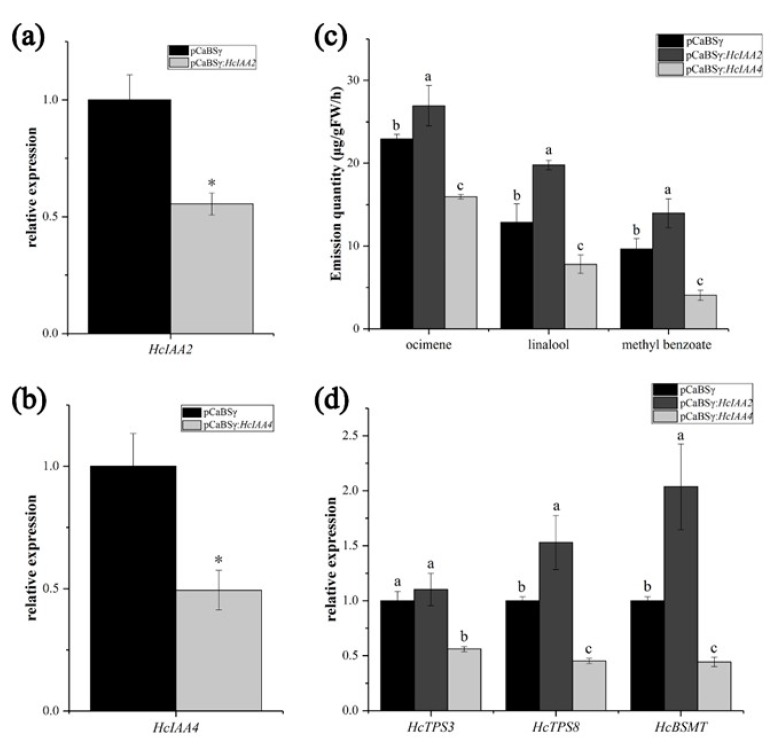
Silencing of *HcIAA2* and *HcIAA4* alters the amount of the main volatile compounds in flowers. (**a, b**) Expression of the *HcIAA2* and *HcIAA4* genes in barley stripe mosaic virus (BSMV)-*HcIAA2/4*-silenced and control flowers were analyzed by qRT-PCR. (**c**) GC–MS analysis of main volatile compounds of *H. coronarium* flower. (**d**) Expression analysis of key volatile biosynthesis genes in BSMV-*HcIAA2/4*-silenced vs. control flowers by qRT-PCR. The results are the means of three biological replicates with standard deviations. Asterisks indicate statistically significant differences (Student’s *t*-test, * *p* < 0.05).

To investigate the potential function of *HcIAA2* and *HcIAA4* in floral volatile compound formation, we suppressed their expression levels by virus-induced gene silencing (VIGS). As shown in Figure 9, when *HcIAA2* and *HcIAA4* were silenced in flowers by VIGS, the expression levels of *HcIAA2* and *HcIAA4* were lower in the silenced flowers than in the pCaBS-γ control (Figure 9a,b). *HcIAA2* silencing caused an increase in the amount of the main volatile compounds compared with the control. The contents of ocimene, linalool and methyl benzoate increased by approximately 17.5%, 54.6% and 44.5%, respectively. In contrast, the *HcIAA4*-silenced flowers showed lower volatile compound amounts than the pCaBSγ control. The contents of ocimene, linalool and methyl benzoate decreased to 30.5%, 39.1% and 58.6%, respectively (Figure 9c). In addition, the expression levels of the key volatile compound synthesis genes in *H. coronarium*, such as *HcTPS3*, *HcTPS8* and *HcBSMT*, were analyzed [35]. In *HcIAA2*-silenced flowers, the expression levels of *HcTPS8* and *HcBSMT* were significantly higher than those of the control; however, the expression levels were reduced in *HcIAA4*-silenced flowers (Figure 9d). The results indicated that *HcIAA2* and *HcIAA4* play an imperative role in floral volatile formation in *H. coronarium.*

## 3. Discussion

Auxins have been shown to play a very important role in plant growth and development [43,45,46]. Two protein families, Aux/IAA and ARF, are known to mediate the auxin signaling molecule pathway [25]. With the development of genome sequencing, the *Aux/IAA* gene family has been reported in more than 30 plant species, including 29 genes from *Arabidopsis thaliana*, 17 from *Medicago truncatula*, 27 from *Cucumis sativus*, 26 from *Solanum lycopersicum*, 26 from *V. vinifera,* 35 from *Populus trichocarpa* and 26 from Citrus [47]. Especially in *Arabidopsis*, the functions of *Aux/IAA* genes are quite well understood [17]. However, in *H. coronarium*, there is very little information available on *Aux/IAA* genes. The characterization and expression pattern analysis of *HcIAA* genes unravel the mechanisms behind auxin involvement in flower development and the floral scent formation of *H. coronarium*. In total, 27 *Aux/IAA* genes were identified in *H. coronarium*, which was slightly less than that of model plants, such as *Arabidopsis* (29) and rice (31). However, the *H. coronarium Aux/IAA* gene family contains a lower number of genes compared with *Arabidopsis*, and two groups are considerably expanded. Groups B and F contain seven and five *Aux/IAA* genes in *H. coronarium*, respectively, but only three members were present in both groups of *Arabidopsis*. Group expansion has also been found in other higher plants, such as *S. lycopersicum* and *P. trichocarpa* [37,48]. Particularly, group H, comprising three non-canonical members (AtIAA20, AtIAA30 and AtIAA31) in *Arabidopsis* that lack the conserved domain II, which is important for protein degradation, is also represented in *H. coronarium* with one member (HcIAA27). Aux/IAA proteins classified into the same groups may have similar functions in events common to both monocot and dicot plants [49]. The results of phylogenetic analyses of *H. coronarium* Aux/IAA proteins will pave the way for their functional analysis.

The promoter analysis revealed several well-identified hormone response elements, including the well-conserved AuxREs, present in the promoter regions of the majority of *HcIAA* genes (Figure 4). Different *cis*-elements in the promoters of *HcIAA* genes partly show that auxin signaling transduction can interact with other metabolic pathways. Furthermore, our results revealed that ethylene responsive elements were enriched in most promoters of *HcIAA* genes (18 out of 27), suggesting that auxin and ethylene play key roles through cross-talk via *HcIAAs* in *H. coronarium* flowers; this result is similar to that found in tomato, in which the same pattern was reported, and 16 out of the 25 *Sl-IAA* promoters contained ethylene-response motifs [37]. This result indicated that *Aux/IAA* genes might also be regulated by ethylene in *H. coronarium* flowers.

The expression profiles of *HcIAA* genes in different tissues and organs disclosed that some *Aux/IAAs* have preferential expression patterns. Though with structural similarity, their expression patterns showed tissue or organ specificity, suggesting the functional diversity of gene families in different biological processes. For example, HcIAA2 and HcIAA15 have 79.6% identity, but the expression patterns were not the same. *HcIAA2* showed much higher mRNA accumulation in flowers, whereas *HcIAA15* was preferentially expressed in the leaves (Figure 5c). From the 27 *HcIAAs*, seven members had high expression levels in the vegetative organs; however, 14 *Aux/IAAs* showed higher expression levels in the flowers. *HcIAA15* and *HcIAA11* showed higher transcript accumulation in the leaves, indicating their functional role in leaf development. Some *HcIAA* genes have temporal and spatial expression patterns, such as *HcIAA2*, *HcIAA4*, *HcIAA16* and *HcIAA22*, which were specifically expressed in the labellum and lateral staminode, the main tissues for the emission of volatile compounds, indicating their important roles in floral scent formation. In *H. coronarium*, the flower volatile compounds start to release during the flower bud stage (S1), then increase at the half-open stage and peak at the full-bloom stage, decreasing in the senescence stage. Interestingly, the expression pattern of Group III *HcIAAs* showed stage-specific expression patterns during the flower developmental stages that were similar to the amount of volatile compounds during the flower developmental stages. This result indicated that Group III members are potentially involved in floral scent formation. Tissue-specific and developmental stage-specific expression manners of *Aux/IAA* genes have been demonstrated in many species, such as chickpea, soybean, maize and cotton [26,50,51]. In the tomato flower development process, from the flower bud to fully open stages, a high mRNA gradient level of *SlIAA9* was established and played a key role in regulating the initiation of fruit set [49]. In chickpea, during flower development, the *Aux/IAA* genes, such as *CaIAA4*, *CaIAA7*, *CaIAA8*, *CaIAA10* and *CaIAA13*, also revealed higher transcript accumulation during the full-bloom stage and then declined at the senescence stage, suggesting their possible role in flower development. For soybean, *GmIAA6*, *GmIAA31* and *GmIAA33* exhibited higher transcript levels in floral buds, suggesting their putative role in the development of flower buds [51]. In cotton, the expression levels of *GhAuxs* showed different patterns during fiber initiation and development stages, contributing to the development of fiber [49]. The stage-specific differential expression suggests the diverse and overlapping functions of these proteins during plant growth and development. Overall, the tissue-preferential and stage-specific expressions exhibited by several *Aux/IAA* genes in *H. coronarium* flowers suggest their involvement in the biology of specific tissues and flower scent formation.

In agreement with previous reports, our data showed that the transcript levels of many *HcIAA* genes were regulated by auxin treatment (Figure 7b). Among these genes, *HcIAA3*, *HcIAA4*, *HcIAA5* and *HcIAA23* showed higher upregulation under auxin treatment. A similar study has already been identified in *Arabidopsis*, tomato, rice and papaya [8,17,30,37]. *Arabidopsis Aux/IAA* gene family members have also been shown to respond to exogenous IAA in a highly differential fashion with respect to time and dose [52]. In tomato, the transcript levels of 17 out of 19 *Sl-IAA* genes were upregulated by auxin treatment in seedlings, while *Sl-IAA2*, *Sl-IAA3*, *Sl-IAA17* and *Sl-IAA19* were significantly induced [37]. In rice, the transcript levels of the majority of *OsIAA* genes were upregulated by auxin treatment, and the effect was more pronounced on *OsIAA9*, *OsIAA14*, *OsIAA19*, *OsIAA20* and *OsIAA24* [53,54]. Interestingly, the expression levels of *HcIAA3*, *HcIAA4*, *HcIAA6*, *HcIAA7* and *HcIAA12* were dramatically reduced by treatment with the auxin signal inhibitor PCIB. The number of volatile compounds was reduced significantly under the PCIB treatment of *H. coronarium* flowers (Figure 7a), showing the importance of auxin signal transduction in floral scent formation. These results indicated that these genes may play key roles in floral scent formation through their expression levels or accumulated proteins in *H. coronarium* flowers. Additionally, the data showed that the content of volatiles in the flowers and the expression levels of some *HcIAA* genes were induced by ethylene treatment (Figure 7a,d). *Aux/IAA* gene responsiveness to ethylene was first described in late immature green tomato fruit [55]. Recent studies provide a comprehensive analysis of the ethylene regulation of *Aux/IAA* genes, revealing that ethylene clearly induced the expression of some genes. In tomato, the transcript accumulation level of *Sl-IAA29* was most strongly upregulated by ethylene, while *Sl-IAA2*, *Sl-IAA11*, *Sl-IAA17* and *Sl-IAA19* were reduced in etiolated seedlings [38]. In papaya, the expression levels of *CpIAA3*, *CpIAA15a*, *CpIAA15b*, *CpIAA19*, *CpIAA27* and *CpIAA32* were significantly upregulated under 1-aminocyclopropane-1-carboxylic acid (ACC) treatment, an ethylene precursor [30]. In *H. coronarium*, the expression levels of some members were upregulated by auxin and ethylene at the same time, such as *HcIAA4* and *HcIAA6,* which were highly upregulated by both auxin and ethylene (Figure 7b,d). Similarly, *Sl-IAA3* transcript accumulation was positively regulated by auxin and ethylene in tomato seedlings [55]. The effect of auxin and ethylene treatment on the transcript levels of *HcIAA* genes reflects their role in auxin and ethylene cross talk in signal transduction. The potential role of the ethylene-regulated *Aux/IAA* genes in mediating the cross-talk between ethylene and auxin remains to be further studied, particularly during flower volatile formation during flower development.

Based on our data, *HcIAA2*, *HcIAA4*, *HcIAA6* and *HcIAA12* were considered to be suitable candidate genes to regulate flower volatile formation in *H. coronarium*, while *HcIAA2* and *HcIAA4* were chosen for functional characterization by VIGS. *HcIAA2* is the ortholog of the *Arabidopsis* gene *AtIAA7*. The function of *AtIAA7* has been studied through gain-of-function experiments. The *AtIAA7* mutation *axr2-1* conferred late flowering under short daylight in *Arabidopsis* [56]. The silencing of *HcIAA2* in *H. coronarium* flowers did not change the flowering process, but the total volatile contents increased. Interestingly, the total volatile content was decreased in silenced flowers (*HcIAA4*), which was in contrast to *HcIAA2*. Similarly, in suppressed *Sl-IAA15* tomato transgenic plants, the content of some monoterpenes in leaf trichome exudates was significantly reduced [19]. The difference in the contents of the volatiles after the silencing of *HcIAA2* and *HcIAA4* may be because they interact with different proteins, such as ARFs, that have an active or repressive function in the auxin signal response. These results, along with the expression patterns of *HcIAA2* and *HcIAA4*, strongly suggest that *HcIAA2* and *HcIAA4* play important roles in flower scent formation in *H. coronarium*.

Floral scent formation is a complex process that is influenced by photoperiod, temperature and phytohormones [57,58,59,60]. Of these key elements, many studies have indicated that hormone signal transduction plays a pivotal role in floral scent formation [61,62,63,64]. Treatment with exogenous IAA and its signal inhibitor, PCIB, significantly influenced the amount of floral volatiles in *H. coronarium* (Figure 7a). Although *HcIAA* genes act as the primary auxin-responsive genes, their mode of action in floral scents needs to be investigated further. In this study, we identified the *Aux/IAA* family genes, analyzed the gene expression profiles, and selected several genes that were important candidates for further functional characterization. By VIGS, we identified that *HcIAA2* and *HcIAA4* are functionally involved in floral volatile formation. Overall, the information reported here for *HcIAA* genes could assist further investigations related to their functions in floral scent formation and elucidate the complicated auxin signaling transduction cascade.

## 4. Materials and Methods

### 4.1. Plant Material, Growth Conditions and Hormone Treatment

*H. coronarium* was grown in the growth chamber in South China Agricultural University Guangzhou, China under natural light conditions at 26 ± 2 °C with 13 h light and 11 h dark cycles. Plant samples were instantaneously frozen in liquid nitrogen after collected from the growth chamber and stored at –80 °C. For the analysis of tissue-specific expression patterns, three different samples were used: Full-bloom flowers, mature green leaves and healthy rhizomes of two-year-old *H. coronarium* plants (Figure 5d). The flower was divided into four parts, i.e., mixed labellum and lateral staminode, corolla lobes, pistil and stamen (Figure 5d). The flower developmental process was divided into four stages: Bud stage (S1), initial flowering stage (S2), full-bloom flower (S3) and senescence (S4; Figure 6b).

The flowers used for hormone treatment were purchased from the *H. coronarium* cut flower market. After brought back to laboratory, the flowers were immediately cultured in Murashige and Skoog (MS) liquid medium. The cut flowers had a similar flower developmental process as natural flowers (mentioned above). For IAA, 2-(4-chlorophenoxy)-isobutyric acid (PCIB) and abscisic acid (ABA) treatment, the flowers were chosen at the developmental stage between S1 and S2. The flower stems were shortly cut into 40 cm and then placed in sterilized water containing 100 μM IAA, 1.5 mM PCIB and 200 μM ABA for 12 h in a chamber with 14 h daylight and 10 h dark at 25 °C. For ethylene treatment, flowers were incubated with 10 μL/L of ethylene for 12 h in a sealed bottle. The volatile compound analysis was carried out at the full-bloom stage of treated flowers, which were subsequently frozen in liquid nitrogen and stored at –80 °C. *Nicotiana benthamiana*, for subcellular localization experiments, were grown in a growth room at 25 °C with a 12 h daylight and 12 h dark period.

### 4.2. RNA Isolation, cDNA Synthesis and Quantitative Real-Time PCR (qRT-PCR)

Total RNA from different organs/tissues and flower developmental stages was extracted using a HiPure plant RNA mini kit (Magen, Guangzhou, China) according to the manufacturer’s suggestions. In total RNA, genomic DNA contamination was removed by DNase I. The qRT-PCR analysis was executed in an ABI 7500 Fast Real-Time PCR System (Applied Biosystems, Waltham, MA, USA) using iTaq™ Universal SYBR Green Supermix (BIO-RAD, Hercules, CA, USA) following the manufacturer’s protocols. PCR was performed in a total volume of 20 μL comprising cDNA, iTaq™ Universal SYBR^®^ Green Supermix and forward and reverse primer. The temperature conditions were as follows: Initial temperature 95 °C for one min followed by 40 cycles of 95 °C for 15 s and 55 °C for 30 s, concluding at 72 °C for 30 s. The relative expression level of genes was calculated according to the formula 2^−ΔΔ*C*t^ method [65]. A similar procedure was carried out with different treatments, and the sequence-specific primers used for qRT-PCR are listed in Table S1. A heat map was constructed to visualize the qRT-PCR data by the Dual System Plotter software.

### 4.3. Genome-Wide Identification of HcIAA Genes

Based on the transcriptome data [29] and genomic data (data unpublished) of *H. coronarium,* which was obtained from the Beijing Novogene Bioinformatics Technology Corporation (China), *HcIAA* genes were identified using 29 *Arabidopsis* Aux/IAA protein sequences. These *Arabidopsis* Aux/IAA protein sequences were used to search for related proteins predicted in the *H. coronarium* genomic data (data unpublished), and 35 potential Aux/IAA proteins were identified. Afterward, the Pfam database Aux/IAA domain (PF02309) and NCBI conserved domain database web servers were used to observe the conserved domains. The incorrectly predicted and redundant sequences were manually discarded. A total of 27 Aux/IAA genes were finally identified in the *H. coronarium* genome. The obtained sequences were identified as unique genes for comprehensive analysis. The basic physical and chemical parameters of the *H. coronarium Aux/IAA* genes were calculated by the online ProtParam tool. Sequence information of *H. coronarium Aux/IAA* genes is given in the Table S3.

### 4.4. Phylogenetic Tree Construction, Gene Structure and Motif Prediction

Multiple sequence alignment of full-length sequences of HcIAA was performed using Clustal X 2.1. A phylogenetic tree was constructed with the aligned AtIAA and HcIAA protein sequences using MEGA6 [66] and choosing the neighbor-joining method. The predictions of the four classical domains (I, II, III and IV) in HcIAA proteins were performed with the DNAMAN software. The DNA and cDNA sequences corresponding to each predicted gene were obtained from the *H. coronarium* genome, and the exon–intron organization of the *HcIAA* genes was analyzed by the Gene Structure Display Server (GSDS). To identify conserved motifs in HcIAA proteins, the Multiple Expectation Maximization for Motif Elicitation (MEME) online program was used for protein sequence analysis. The optimized parameters were designed as follows: The incidences of a single motif 0 or 1 per sequence, motif width ranges from 10 to 60 amino acids, the maximum number of motifs to find four and other parameters defaulted.

### 4.5. Analysis of Hormone-Related cis-Elements

To investigate *cis*-elements in the promoter sequences of *H. coronarium Aux/IAA* genes, sequences 2000 bp upstream of the initiation codon were selected from the *H. coronarium* genome database. Numerous hormone-related *cis*-elements were analyzed, including auxin-responsive element (AuxRE), ethylene-responsive element (ERE), ABA-responsive element (ABRE), SA-responsive element (SARE) and gibberellin-responsive element (GARE). Moreover, to identify the putative cis-regulatory elements along the promoter sequences of each HcIAA family gene, the PLACE website was used [67].

### 4.6. Subcellular Localization of HcIAA Genes

The open reading frame (ORFs) of HcIAA2, HcIAA4, HcIAA6 and HcIAA12 were fused into the vector pEAQ-HT-GFP [68] using the *Age* I enzyme at the restriction site. The ClonExpress^®^ II one step cloning kit (Vazyme, China) was used to construct the vectors. Sequencing confirmed that no errors had been introduced. The primers used in this experiment are listed in Table S1. The combined plasmids were introduced into *Agrobacterium tumefaciens* (strain EHA105). The cultures were grown in Luria–Bertani (LB) medium with antibiotics and shaken overnight to stationary phase. The following day, pellets were collected by centrifugation at 2000 g and resuspended in MMA (10 mM MgCl_2_, 100 µM acetosyringone, 10 mM MES (2-[N-morpholino] ethane sulfonic acid) with pH 5.8 to an OD_600_ of 0.6 for 2-3 h incubation at room temperature. The suspensions were infiltrated into *N. benthamiana* leaves. The infected tissues were visualized 48 h after infiltration by a Leica TCS SP2 AOBS Spectral Confocal Scanner mounted on a Leica DM RXA2 upright fluorescence microscope with 409 × 0.75 numerical aperture objectives, and images were further processed using Adobe Photoshop.

### 4.7. Headspace Analysis of Floral Volatiles

For volatile analysis, the whole flower was enclosed in a 500 mL glass bottle supplemented with an internal standard. After 30 min, a PDMS (polydimethylsiloxane) fiber was inserted into to adsorb volatiles for 30 min followed by injection into a gas chromatography–mass spectrometry (GC–MS) system (Agilent) for volatile analysis as described previously [35].

### 4.8. Virus-Induced Gene Silencing (VIGS)

The barley stripe mosaic virus (BSMV) system was selected and successfully applied in monocots for virus-induced gene silencing [69,70,71]. pCaBS-α, pCaBS-β and pCaBSγ are essential components of the BSMV system and they ensure the high-efficiency infectivity and transformation of the virus in the cell [71]. The Agro/LIC BSMV-VIGS vectors used in this experiment were provided by Dr. Dawei Li (State Key Laboratory of Agro-Biotechnology, China Agricultural University, Beijing, People’s Republic of China). The empty vector pCaBSγ was linearized with *Apa* I to insert the fragments. At the 3′ end of *HcIAA2* and *HcIAA4*, 280 bp fragments were amplified by PCR from *H. coronarium* cDNAs, especially to silence *HcIAA2* and *HcIAA4*. The fragments were then inserted into the pCaBSγ empty vector to generate the pCaBSγ:*HcIAA2* and pCaBSγ:*HcIAA4* constructs. The primers used for amplifying *HcIAA2* and *HcIAA4* are listed in Table S1**.** The pCaBS-α [71], pCaBS-β [71], pCaBSγ [71], pCaBSγ:*HcIAA2* and pCaBSγ:*HcIAA4* vectors were transformed into *A. tumefaciens* strain EHA105. The transformed *A. tumefaciens* lines were cultured in LB medium supplemented with 50 µg/mL kanamycin and 25 µg/mL rifampicin. The cultures were harvested by centrifugation at 5000 rpm for 10 min and resuspended in infiltration buffer (10 mM MgCl_2_, 0.1 mM acetosyringone, 10 mM MES, pH 5.6) to a final OD_600_ of approximately 1.0. Mixtures of cultures containing an equal ratio (v/v/v) of pCaBS-α, pCaBS-β and pCaBSγ, or pCaBS-α, pCaBS-β and pCaBSγ:*HcIAA2*, or pCaBS-α, pCaBS-β and pCaBSγ:*HcIAA4*. The culture mixtures were placed at room temperature in the dark for 3 to 5 h before vacuum infiltration into the flowers. For VIGS, the flowers were collected at the S1 stage. Vacuum infiltration was carried out by immersing the flowers in the bacterial suspension. After the release of the vacuum, the flowers were washed in deionized water, placed into an MS medium liquid culture, and then maintained with a 12/12 h day/night cycle at 16 °C for five days. The total volatile compounds were collected and analyzed at the full-bloom stage by GC–MS as described above. The experiment was replicated three times.

### 4.9. Statistical Analysis

Statistical analysis was performed using the SPSS 19.0 program (SPSS Inc. Chicago, IL, USA). Comparisons between two groups were executed by using a Student’s *t*-test at a significance level of 0.05. All data are presented as the mean, SD, and *p* < 0.05 was considered statistically significant. The expression analyses were performed between three to four biological replicates.

### 4.10. Data Availability

All data generated or analyzed during this study are included in the main text or supplement of this published article.

## Figures and Tables

**Table 1:** Description of the *Aux/IAA* gene family in *Hedychium coronarium.*

Gene Name	Gene ID	ORF (bp)	Deduced Polypetide			Exon No.	Genome Location
			Length (aa)	Mol. Wt (kda)	pI		
*HcIAA1*	Hc118.198	552	183	20.698	9.13	5	1298266–1301736
*HcIAA2*	Hc909.36	735	244	26.869	8.65	6	311277–314281
*HcIAA3*	Hc563.22	501	166	19.177	4.84	4	170726–171832
*HcIAA4*	Hc215.30	855	284	31.179	9.04	6	354002–357876
*HcIAA5*	Hc1096.12	891	296	31.617	5.09	4	96290–98064
*HcIAA6*	Hc506.60	594	197	21.856	8.57	4	520699–522248
*HcIAA7*	Hc717.7	468	155	16.881	9.36	3	53508–54653
*HcIAA8*	Hc38.101	852	283	30.625	7.42	5	1966779–1969857
*HcIAA9*	Hc899.17	924	307	32.633	7.01	5	251006–254190
*HcIAA10*	Hc357.107	945	314	33.141	6.16	5	779004–783999
*HcIAA11*	Hc189.134	777	258	28.457	5.97	4	1076907–1077976
*HcIAA12*	Hc189.121	594	197	21.273	5.30	3	987719–988944
*HcIAA13*	Hc107.22	912	303	33.216	9.31	5	734366–735832
*HcIAA14*	Hc285.12	735	244	26.556	7.93	5	146079–148497
*HcIAA15*	Hc440.26	729	242	26.643	9.23	5	424147–425365
*HcIAA16*	Hc47.162	528	175	19.225	5.11	4	1790499–1791855
*HcIAA17*	Hc1004.1	537	178	20.500	4.66	3	64402–73225
*HcIAA18*	Hc412.59.2	933	310	34.252	5.91	6	489469–493695
*HcIAA19*	Hc269.7	1005	334	35.863	8.23	5	386761–389599
*HcIAA20*	Hc41.21	942	313	33.433	6.55	5	707930–711511
*HcIAA21*	Hc614.34	858	285	30.632	6.55	5	348817–352993
*HcIAA22*	Hc42.52	585	194	21.522	5.40	5	737421–740882
*HcIAA23*	Hc270.58	615	204	23.116	10.15	3	459106–460132
*HcIAA24*	Hc484.51.1	1059	352	39.190	6.29	7	480251–483666
*HcIAA25*	Hc566.42	546	181	19.907	5.45	4	628269–630501
*HcIAA26*	Hc641.57	762	253	27.553	7.14	5	340256–342758
*HcIAA27*	Hc326.19	522	173	19.566	6.78	4	329556–335070

## Supplementary Materials

The supplementary materials are available online at https://www.mdpi.com/1422-0067/20/13/3235/s1.

## Abbreviations

Aux/IAA	Auxin/indole-3-acetic acid
SAUR	Small Auxin UP RNA
GH3	Gretchen Hagen 3
ARF	Auxin response factor
AuxREs	Auxin-responsive *cis*-elements
MS	Murashige and Skoog
ABA	Abscisic acid
PCIB	2-(4-chlorophenoxy)-isobutyric acid
IAA	Indole-3-Acetic Acid
GSDS	Gene Structure Display Server
MEME	Multiple Expectation Maximization for Motif Elicitation
AuxRE	Auxin-responsive Element
ERE	Ethylene-responsive element
GARE	Gibberellin-responsive element
SARE	SA-responsive element
ABRE	ABA-responsive element
ORFs	Open reading frame
LB	Luria-Bertani
PDMS	Polydimethylsiloxane
GC-MS	Gas chromatography-mass spectrometry
VIGS	Virus-induced gene silencing
BSMV	Barley stripe mosaic virus
MW	Molecular weight
pI	Isoelectric point
qRT-PCR	Quantitative real-time polymerase chain reaction
GFP	Green fluorescence protein

## References

[1] Hagen G., Guilfoyle T. (2002). Auxin-responsive gene expression: Genes, promoters and regulatory factors. Plant Mol. Biol..

[2] Friml J., Vieten A., Sauer M., Weijers D., Scwartz H., Hamann T., Offringa R., Jürgens G. (2003). Efflux-dependent auxin gradients establish the apical-basal axis of Arabidopsis. Nature.

[3] Jain M., Kaur N., Garg R., Thakur J.K., Tyagi A.K., Khurana J.P. (2006). Structure and expression analysis of early auxin-responsive *Aux/IAA* gene family in rice (*Oryza sativa*). Funct. Integr. Genom..

[4] Theologis A., Huynh T.V., Davis R.W. (1985). Rapid induction of specific mRNAs by auxin in pea epicotyl tissue. J. Mol. Biol..

[5] Oeller P.W., Keller J.A., Parks J.E., Silbert J.E., Theologis A. (1993). Structural characterization of the early indoleacetic acid-inducible genes, PS-IAA4/5 and PS-IAA6, of pea (*Pisum sativum* L.). J. Mol. Biol..

[6] Dharmasiri S., Estelle M. (2002). The role of regulated protein degradation in auxin response. Plant Mol. Biol..

[7] Tiwari S.B., Wang X.J., Hagen G., Guilfoyle T.J. (2001). Aux/IAA proteins are active repressors, and their stability and activity are modulated by auxin. Plant Cell.

[8] Abel S., Oeller P.W., Theologis A. (1994). Early auxin-induced genes encode short-lived nuclear proteins. Proc. Natl. Acad. Sci. USA.

[9] Arase F., Nishitani H., Egusa M., Nishimoto N., Sakurai S., Sakamoto N., Kaminaka H. (2012). *IAA8* involved in lateral root formation interacts with the TIR1 auxin receptor and ARF transcription factors in Arabidopsis. PLoS ONE.

[10] Tiwari S.B. (2004). Aux/IAA proteins contain a potent transcriptional repression domain. Plant Cell.

[11] Szemenyei H., Hannon M., Long J.A. (2008). TOPLESS Mediates Auxin-Dependent Transcriptional Repression During *Arabidopsis* Embryogenesis. Science.

[12] Dharmasiri N., Dharmasiri S., Estelle M. (2005). The F-box protein TIR1 is an auxin receptor. Nature.

[13] Kepinski S., Leyser O. (2005). The *Arabidopsis* F-box protein TIR1 is an auxin receptor. Nature.

[14] Tan X., Calderon-Villalobos L.I.A., Sharon M., Zheng C., Robinson C.V., Estelle M., Zheng N. (2007). Mechanism of auxin perception by the TIR1 ubiquitin ligase. Nature.

[15] Leyser O. (2018). Auxin Signaling. Plant Physiol..

[16] Remington D.L. (2004). Contrasting modes of diversification in the Aux/IAA and ARF gene families. Plant Physiol..

[17] Overvoorde P.J. (2005). Functional genomic analysis of the Auxin/indole-3-acetic acid gene family members in *Arabidopsis thaliana*. Plant Cell.

[18] Tiwari S.B. (2003). The Roles of Auxin Response Factor Domains in Auxin-Responsive Transcription. Plant Cell..

[19] Deng W., Yan F., Liu M., Wang X., Li Z. (2014). Down-regulation of *SlIAA15* in tomato altered stem xylem development and production of volatile compounds in leaf exudates. Plant Signal. Behav..

[20] Bassa C., Mila I., Bouzayen M., Audran-Delalande C. (2012). Phenotypes associated with down-regulation of *Sl-IAA27* support functional diversity among *AUX/IAA* family members in tomato. Plant Cell Physiol..

[21] Guillotin B., Etemadi M., Audran C., Bouzayen M., Bécard G., Combier J. (2016). *Sl-IAA27* regulates strigolactone biosynthesis and mycorrhization in tomato (var. Micro Tom). New Phytol..

[22] Woodward A.W., Bartel B. (2005). Auxin: Regulation, action, and interaction. Ann. Bot..

[23] Zhu B.Q., Xu X.Q., Wu Y.W., Duan C.Q., Pan Q.H. (2012). Isolation and characterization of two hydroperoxide lyase genes from grape berries: HPL isogenes in *Vitis vinifera* grapes. Mol. Biol. Rep..

[24] Kitomi Y., Inahashi H., Takehisa H., Sato Y., Inukai Y. (2012). OsIAA13-mediated auxin signaling is involved in lateral root initiation in rice. Plant Sci..

[25] Liscum E., Reed J.W. (2002). Genetics of Aux/IAA and ARF action in plant growth and development. Plant Mol. Biol..

[26] Wang Y., Deng D., Bian Y., Lv Y., Xie Q. (2010). Genome-wide analysis of primary auxin-responsive *Aux/IAA* gene family in maize (*Zea mays.* L.). Mol. Biol. Rep..

[27] Wu J., Peng Z., Liu S.Y., He Y.J., Cheng L., Kong F., Wang J., Lu G. (2012). Genome-wide analysis of Aux/IAA gene family in *Solanaceae* species using tomato as a model. Mol. Genet. Genom..

[28] Çakir B., Kiliçkaya O., Olcay A. (2013). Genome-wide analysis of Aux/IAA genes in *Vitis vinifera*: Cloning and expression profiling of a grape *Aux/IAA* gene in response to phytohormone and abiotic stresses. Acta Physiol. Plant..

[29] Yue Y., Yu R., Fan Y. (2015). Transcriptome profiling provides new insights into the formation of floral scent in *Hedychium coronarium*. BMC Genom..

[30] Liu K., Yuan C., Feng S., Zhong S., Li H., Zhong J., Shen C., Liu J. (2017). Genome-wide analysis and characterization of *Aux/IAA* family genes related to fruit ripening in papaya (*Carica papaya* L.). BMC Genom..

[31] Wu Z.Y., Raven H.P. (2000). Flora of China.

[32] Baez D., Pino J.A., Morales D. (2011). Floral scent composition in *Hedychium coronarium* J. Koenig analyzed by SPME. J. Essent. Oil Res..

[33] Fan Y.P., Yu R.C., Huang Y., Chen Y.F. (2003). Studies on the essential constituent of *Hedychium flavum* and *H. coronarium*. Acta Horti. Sinica.

[34] Fan Y.P., Wang X.R., Yu R.C., Yang P. (2007). Analysis on the aroma components in several species of *Hedychium*. Acta Horti. Sinica.

[35] Yue Y., Yu R., Fan Y. (2014). Characterization of two monoterpene synthases involved in floral scent formation in *Hedychium coronarium*. Planta.

[36] Pichersky E., Dudareva N. (2007). Scent engineering: Toward the goal of controlling how flowers smell. Trends Biotechnol..

[37] Audran-Delalande C., Bassa C., Mila I., Regad F., Zouine M., Bouzayen M. (2012). Genome-Wide identification, functional analysis and expression profiling of the *Aux/IAA* gene family in tomato. Plant Cell Physiol..

[38] Wils C.R., Kaufmann K. (2017). Gene-regulatory networks controlling inflorescence and flower development in *Arabidopsis thaliana*. Biochim. Biophys. Acta Gene Regul. Mech..

[39] Zubo Y.O., Yamburenko M.V., Kusnetsov V.V., Börner T. (2011). Methyl jasmonate, gibberellic acid, and auxin affect transcription and transcript accumulation of chloroplast genes in barley. J. Plant Physiol..

[40] Agarwal P.K., Agarwal P., Custers J.B.M., Liu C., Bhojwani S.S. (2006). PCIB an anti-auxin enhances microspore embryogenesis in microspore culture of *Brassica juncea*. Plant Cell Tissue Organ Cult..

[41] Fransson P. (1958). Studies on the interaction of antiauxin and native auxin in wheat roots. Physiol. Plantarum.

[42] Heupel T., Stange L. (1995). The auxin antagonist *p*-chlorophenoxyisobutyric acid abolishes polar distribution of DNA synthesizing cells within the meristem of *Riella helicophylla*. J. Plant Physiol..

[43] Van-Doorn W.G., Dole I., Çelikel F.G., Harkema H. (2013). Opening of Iris flowers is regulated by endogenous auxins. J. Plant Physiol..

[44] Yu H., Soler M., San Clemente H., Mila I., Paiva J.A.P., Myburg A.A., Bouzayen M., Grima-Pettenati J., Cassan-Wang H. (2015). Comprehensive genome-wide analysis of the *Aux/IAA* gene family in *Eucalyptus*: Evidence for the role of *EgrIAA4* in wood formation. Plant Cell Physiol..

[45] Tyurin A.A., Kabardaeva K.V., Berestovoy M.A., Sidorchuk Y.V., Fomenkov A.A., Nosov A.V., Goldenkova-Pavlova I.V. (2017). Simple and reliable system for transient gene expression for the characteristic signal sequences and the estimation of the localization of target protein in plant cell. Russ. J. Plant Physiol..

[46] Bohn-Courseau I. (2010). Auxin: A major regulator of organogenesis. Comptes Rendus Biologies.

[47] Gaudinová A., Malbeck J., Dobrev P., Kubelková D., Špak J., Vanková R. (2008). Cytokinin, auxin, and abscisic acid dynamics during flower development in white and red currants infected with Black currant reversion virus. Physiol. Mol. Plant Pathol..

[48] Luo J., Zhou J., Zhang J. (2018). *Aux/IAA* Gene Family in Plants: Molecular Structure, Regulation, and Function. Int. J. Mol. Sci..

[49] Kalluri U.C., Difazio S.P., Brunner A.M., Tuskan G.A. (2007). Genome-wide analysis of *Aux/IAA* and *ARF* gene families in (*Populus trichocarpa*). BMC Plant Biol..

[50] Wang J., Yan D., Yuan T., Gao X., Lu Y. (2013). A gain-of-function mutation in *IAA8* alters *Arabidopsis* floral organ development by change of jasmonic acid level. Plant Mol. Biol..

[51] Han X., Xu X., Fang D.D., Zhang T., Guo W. (2012). Cloning and expression analysis of novel *Aux/IAA* family genes in *Gossypium hirsutum*. Gene.

[52] Singh V.K., Jain M. (2015). Genome-wide survey and comprehensive expression profiling of *Aux/IAA* gene family in chickpea and soybean. Front. Plant Sci..

[53] Reed J.W. (2001). Roles and activities of Aux/IAA proteins in *Arabidopsis*. Trends Plant Sci..

[54] Jain M., Kaur N., Tyagi A.K., Khurana J.P. (2006). The auxin-responsive GH3 gene family in rice (*Oryza sativa*). Funct. Integr. Genom..

[55] Meir S., Philosoph-Hadas S., Sundaresan S., Selvaraj K.S.V., Burd S., Ophir R., Kochanek B., Reid M.S., Jiang C.Z., Lers A. (2010). Microarray analysis of the abscission-related transcriptome in the tomato flower abscission zone in response to auxin depletion. Plant Physiol..

[56] Chaabouni S., Jones B., Delalande C., Wang H., Li Z., Mila I., Frasse P., Latché A., Pech J., Bouzayen M. (2009). *Sl-IAA3*, a tomato *Aux/IAA* at the crossroads of auxin and ethylene signaling involved in differential growth. J. Exp. Bot..

[57] Mai Y.X., Wang L., Yang H.Q. (2011). A gain-of-function mutation in IAA7/AXR2 confers late flowering under short-day light in Arabidopsis. J. Integr. Plant Biol..

[58] Abbas F., Ke Y., Yu R., Yue Y., Amanullah S., Jahangir M.M., Fan Y. (2017). Volatile terpenoids: Multiple functions, biosynthesis, modulation and manipulation by genetic engineering. Planta.

[59] Abbas F., Ke Y., Yu R., Fan Y. (2019). Functional characterization and expression analysis of two terpene synthases involved in floral scent formation in *Lilium ‘Siberia’*. Planta.

[60] Dudareva N., Negre F., Nagegowda D.A., Orlova I. (2006). Plant volatiles: Recent advances and future perspectives. Crit. Rev. Plant Sci..

[61] Dudareva N., Klempien A., Muhlemann J.K., Kaplan I. (2013). Biosynthesis, function and metabolic engineering of plant volatile organic compounds. New Phytol..

[62] Kwangjin K., Mijung K., Heungdeug K., Song J.S., Eunha Y., Junggun C. (2006). The effect of flower scent and essential oils on reduction of concentration of cortisol, a stress hormone. Korean J. Hortic. Sci..

[63] Ramya M., Kwon O.K., An H.R., Park P.M., Baek Y.S., Park P.H. (2017). Floral scent: Regulation and role of MYB transcription factors. Phytochem. Lett..

[64] Schmelz E.A., Engelberth J., Alborn H.T., Donnell P., Sammons M., Toshima H., Tumlinson J.H. (2003). Simultaneous analysis of phytohormones, phytotoxins, and volatile organic compounds in plants. Proc. Natl. Acad. Sci. USA.

[65] Livakm K.J., Schmittgen T.D. (2001). Analysis of relative gene expression data using real-time quantitative PCR and the 2^-ΔΔCt^ method. Methods.

[66] Tamura K., Peterson D., Peterson N., Stecher G., Nei M., Kumar S. (2013). MEGA5: Molecular evolutionary genetics analysis using maximum likelihood, evolutionary distance, and maximum parsimony methods. Mol. Biol. Evol..

[67] Higo K., Ugawa I.M., Korenaga T. (1999). Plant cis-acting regulatory DNA elements (PLACE) database: 1999. Nucleic Acids Res..

[68] Sainsbury F., Thuenemann E.C., Lomonossoff G.P. (2009). pEAQ: Versatile expression vectors for easy and quick transient expression of heterologous proteins in plants. Plant Biotechnol. J..

[69] Mahadevan C., Jaleel A., Deb L., Thomas G., Sakuntala M. (2014). Development of an efficient virus induced gene silencing strategy in the non-model wild ginger *Zingiber zerumbet* and investigation of associated proteome changes. PLoS ONE.

[70] Renner T., Bragg J., Driscoll H.E., Cho J., Jackson A.O., Specht C.D. (2009). Virus-Induced Gene Silencing in the Culinary Ginger (*Zingiber officinale*): An effective mechanism for down-regulating gene expression in tropical monocots. Mol. Plant.

[71] Yuan C., Li C., Yan L., Jackson A.O., Liu Z., Han C., Yu J., Li D. (2011). A high throughput barley stripe mosaic virus vector for virus induced gene silencing in monocots and dicots. PLoS ONE.

