# Supplementary material for: Genome-Wide Analysis and Characterization of the Aux/IAA Family Genes Related to Floral Scent Formation in Hedychium coronarium
Source: Int J Mol Sci. 2019 Jul 1;20(13):3235. doi: 10.3390/ijms20133235 (PMC6651449; doi:10.3390/ijms20133235)
Supplement: Supplementary file 1 [file ijms-20-03235-s001.zip › ijms-514365 supp final/ijms-514365 supp/Supplementary Table S1.docx]

**Supplement Table S1. Primers used in this study**

| Primers name | Forward primer (5’-3’) | Reverse primer(5’-3’) | Notes |
| --- | --- | --- | --- |
| ***HcIAA1*** | GC GTTTCACCATTGGGCATTG | G CATCACCAACAAGCATCCA | qRT-PCR |
| ***HcIAA2*** | GGCTCTCCTCCTTCACCATTT | GGATCCCCATCCTTGTCTTC | qRT-PCR |
| ***HcIAA3*** | GCTATGAGGACGAAGAAGGG | GG CTTTCCTTCTGCTTCATCAG | qRT-PCR |
| ***HcIAA4*** | GCGTTTCCACTGCCAAGAGA | GGCCAAAGTAAGCACACACGAT | qRT-PCR |
| ***HcIAA5*** | AGACGGGGACTGGATGCTG | GCGTATTAATCCGCTCGCCTT | qRT-PCR |
| ***HcIAA6*** | GCATGAAAGGGTATGAAGCG | GCCGTAGTAAATCAATCCCAAC | qRT-PCR |
| ***HcIAA7*** | GGTTGCAAAGGCTCAGACTA | GTTGACTCA GACCCTCCTC | qRT-PCR |
| ***HcIAA8*** | ATGCTTGTTGGTGATGTGC | AGCTTCTGATTCATGTGCG | qRT-PCR |
| ***HcIAA9*** | GGC TCCATCAAAGAACAAAGAA | GCC TAGTGCCAATGAGAACTCC | qRT-PCR |
| ***HcIAA10*** | GG GAAGATGCTGATGGAAAGC | CC CAATGGTGAAGCCACTAAA | qRT-PCR |
| ***HcIAA11*** | G GTAATCCCCACAGTGAAGC | GCG TTAATTGCTGAAGTAAAC | qRT-PCR |
| ***HcIAA12*** | ATGATGCCGATGCGAAGC | CCACACTGGCCGAAGGTG | qRT-PCR |
| ***HcIAA13*** | GCG ACCCATTGGAAGAAAAGTA | GC ATCTCGTCCCCTTCATCGT | qRT-PCR |
| ***HcIAA14*** | GGCTCCTTCACCAACAATG | G GCATCCAATCTCCATCCTT | qRT-PCR |
| ***HcIAA15*** | C GTTCTCCTCCTTCGCCAC | CGC ACCTACGAGCATCCAGTC | qRT-PCR |
| ***HcIAA16*** | GCCTTGGACAGCATGTTCAATTGC | GATGTAAACATCTCCCAAGG | qRT-PCR |
| ***HcIAA17*** | GGTGGACCTCTCCCTTCAT | GCCCATCCTCATCCTCATAAG | qRT-PCR |
| ***HcIAA18*** | CGAATGAATGAGGAAGCAA | GC AAACAAACATGTCCCAAGG | qRT-PCR |
| ***HcIAA19*** | CCG ACCCTCACTAACACTGTCC | AAGCATCCAATCACCATCC | qRT-PCR |
| ***HcIAA20*** | GGCTTGGGATGCCTTTACG | GCTGGTGAAGCCACTAAACAT | qRT-PCR |
| ***HcIAA21*** | CC AGGGGTGGAATGCCTCTAT | GCC ATCACTCGACCCTCTGTTA | qRT-PCR |
| ***HcIAA22*** | TGCCCGCAATTATCCCAAT | ATCACCGACAAGCATCCAA | qRT-PCR |
| ***HcIAA23*** | CC CAAGGGCTACAAGGAACTA | G CTCCAACGAGCATCAAATC | qRT-PCR |
| ***HcIAA24*** | GCG GATGAGGAAGCAAAAAGAG | GC GCAGTAGTCGAGCTGAAAG | qRT-PCR |
| ***HcIAA25*** | G TGGATGGAGCTCCTTACTT | G GCACCCTTCTTTTCTTGTC | qRT-PCR |
| ***HcIAA26*** | CTCCTTCACCACCAGCACT | CATCACCGACAAGCATCCA | qRT-PCR |
| ***HcIAA27*** | GAAACTCGACTTGTTCGCG | GAAACTCGACTTGTTCGCG | qRT-PCR |
| **HcIAA2-GFP** | CAAATTCGCGACCGGTATGATGGCTAGTGGATTG | TGCTAGTCATACCGGTGCTTCTGTTCTTACGTT | GFP |
| **HcIAA4-GFP** | CAAATTCGCGACCGGTATGGCGGAGGAGAAAAAG | TGCTAGTCATACCGGTTGCCACAACATTGAAGT | GFP |
| **HcIAA6-GFP** | CAAATTCGCGACCGGTATGGAGACTGCCTTGGGT | TGCTAGTCATACCGGTTGTTTAGACTGTAATCCTC | GFP |
| **HcIAA12-GFP** | CAAATTCGCGACCGGTATGACGCCGCCAATGGAGC | TGCTAGTCATACCGGTGCCGACGAGCATCCAATC | GFP |
| **HcIAA2-BSMV** | AAGGAAGTTTAAGAATCCCCGAACCAGAACTC | AACCACCACCACCGTACCTGAACCATCTAATAAGCC | VIGS |
| **HcIAA4-BSMV** | AAGGAAGTTTAA ATGTTCTCCTCCTTCACCATTT | AACCACCACCACCGTGCTTCTGTTCTTACGTTTCTCC | VIGS |
