# Supplementary material for: Genome-Wide Analysis and Characterization of the Aux/IAA Family Genes Related to Floral Scent Formation in Hedychium coronarium
Source: Int J Mol Sci. 2019 Jul 1;20(13):3235. doi: 10.3390/ijms20133235 (PMC6651449; doi:10.3390/ijms20133235)
Supplement: Supplementary file 1 [file ijms-20-03235-s001.zip › ijms-514365 supp final/ijms-514365 supp/Supplementary Table S3.docx]

**HcIAA1 >evm.model.scaf_118.198**

ATGTTGGGACAAAGGCGGCTGGTCCGCGATTCGAAGATCTCCGTGGGCAATTCGGACGAAATCCAGCGTGAAATTGCTCCTGCTGCAAAGGCACAGGTAGTTGGTTGGCCACCAATTCGTAGTTACCGAAAGAACACAATGGCTACTAATCCTCCAAAGAACAAAGAAGTGGATGGAAGACATGGGTTTGGTTGTCTTTATGTCAAGGTTAGCATGGATGGTGCTCCTTATCTCAGGAAAGTTGACTTGATAACATATAGTGACTACAAAGAACTTTCTTTGGCTCTTGAAAAGATGTTCAGTGGTTTCACCATTGGGCATTGCAGCTCTCAAGAAATGAGAGACGGGCTATCTGAAAGCAGGTTGTTGGATCTATTAAATGGATCTGAATATGTCCTTACTTACGAAGACAAGGATGGAGATTGGATGCTTGTTGGTGATGTGCCATGGAAGATGTTCACTGACTCCTGCAGAAGATTGAGGATCATGAAGGGTTCTGATGCAATTGGACTCGCTCCAAGGGCCATGGAGAAGTGCAAGAACCGGAACTAG

**Protein sequence**

MLGQRRLVRDSKISVGNSDEIQREIAPAAKAQVVGWPPIRSYRKNTMATNPPKNKEVDGRHGFGCLYVKVSMDGAPYLRKVDLITYSDYKELSLALEKMFSGFTIGHCSSQEMRDGLSESRLLDLLNGSEYVLTYEDKDGDWMLVGDVPWKMFTDSCRRLRIMKGSDAIGLAPRAMEKCKNRN

**HcIAA2 >evm.model.scaf_909.36**

ATGAAAATAAGACCACTGATGCTCATGAGAAGCCACCTGCCCCCAAAGCACAAGTGGTGGGCTGGCCTCCAGTTCGATCCTTCAGGAAGAACTATTCTTAACGTGCAGCAACATCCTGAGAGCAAGAACAAGGAAGAACAAAGCAAGAAAACAGCAGCTGCTTCAGCCGCCGCCTTCGTGAAGGTGAGCATGGACGGCGCTCCTTACCTTCGCAAGGTCGACCTCAAGACCTACAAGAGCTACCAAGAGCTCTCCAAGGCCTTGCAGAAGATGTTCTCCTCCTTCACCATTTCCGGAAATTATGGATCTGAACAAGGAATAGGTGTGAGGGATTTCATGAACGATGGCAAGGTAACTGAACTGGTGATCAATGGATCTGAGTTTGTGCCAACTTTTGAAGACAAGGATGGGGATTGGATGCTCGTGGGGGACGTTCCTTGGGAGATGTTTGTTGACTCCTGCAAACGCTTGAGGATAATGAAGGGATCAGAGGCCGTCGGACTTGCACCGAGGGCCATGGAGAAACAACCGCGACTTGTCGTCGACTCCCGACGATGGCCGATTGCCGCCGCGTCCTTCTCCACCATCCCTCCCCGTCTCCTTCCTTTTTCCATCTCCCTCCCTGCTCTCCGGCCGACGGATCCCTCCGCCTTGGCGTCCCGCTTCTACGCAGAGCACCGACGTCTCTCCTACCGAGCACCTCCGTCGCGCCGCCTCCTTCCTTTGCCCGTCTCCGTTTCCAAGGTCGTCCTCTCCTTCCTCCGTCGCCTAACCGATCGCGCCGCCGCTCCTAATCCGGAGGAGCTATCTCAGCTTCGCTCACTCCAATGGGACCTGACCAGGGCCTTCGGGCCTCTTTTCTTTTCCGGGGCAGGCGGCCCGGCCGTTAGGCTACCTCAACACCCCGCTCACGGTGGTGGTTGCTGGGATGGCGAAGTGGCTCGACATCTACAGCTGCGTGCTGATGATTAG

**Protein sequence**

MMASGLRLEETELRLGLPGGGSGGCGGEADVSVKSSGKRGFLETIDLKLKLHTPVEDKELETTENENKTTDAHEKPPAPKAQVVGWPPVRSFRKNILNVQQHPESKNKEEQSKKTAAASAAAFVKVSMDGAPYLRKVDLKTYKSYQELSKALQKMFSSFTISGNYGSEQGIGVRDFMNDGKVTELVINGSEFVPTFEDKDGDWMLVGDVPWEMFVDSCKRLRIMKGSEAVGLAPRAMEKRKNRS

**HcIAA3 >evm.model.scaf_563.22**

ATGGAGTTGGAGCTCGGCCTTGCACTCCCCAGCTCTGATCTGATGATGGAGAAGAGTACCTTTTGCAAGAAAAGACGCTTCGATGAGGTGCTCGACGAAAACACTGTTACTTTGCCTCTCTTCATGCAGAGGGAGGAAGACAGCGACAGTGACAGGGGATTTGAGCGTTCTTCGGAGGAGCCTAACTTTGGTGGTGGAGGTAGAGTTATAGTTGGTTGGCCGCCGGTGAAGTTGTCGAGGAGGAGGACGAGCTGCGTGAAGGTGAACATGGAGGGGATTGCCATAGGAAGGAAGGTGGACCTCTCCCTCCATGATTCTTACCAGGCTCTCTTCCTCACACTATACACGATGTTCCCTAAGAAGCATCATGAGGAAGAGGATGGAGCGTATAATTATTACAAAGTGACTTATGAGGACGAAGAAGGGGATTGGATGCTGGTTGGAGATGTGCCATGGGAAGCTTTTATCAAGTCGGCGAAGCGTCTCAAGATAATCAACTGA

**Protein sequence**

MELELGLALPSSDLMMEKSTFCKKRRFDEVLDENTVTLPLFMQREEDSDSDRGFERSSEEPNFGGGGRVIVGWPPVKLSRRRTSCVKVNMEGIAIGRKVDLSLHDSYQALFLTLYTMFPKKHHEEEDGAYNYYKVTYEDEEGDWMLVGDVPWEAFIKSAKRLKIIN

**HcIAA4 >evm.model.scaf_215.30**

ATGGCGGAGGAGAAAAAGCCGCTCGACTGGAGCACCGCGGAGGAGAGGAAGCTAGAGCTAAGGCTTGGCCTCCCCGGAGAAGGAGAGGACTGGTCGCCGTCGCAGGAGAAGGGAAAAAAGCACCTCCCTCTTGAGACCTCCCTTTCCCTCGGGCACGTCTCCAAGGTTCCAAGAAGCAACAATTTCACTGCTTCCTCTGCAACCAAAAGAGAAGTTGTTGCAGCTTTTCAGCAGCCGAAGCAGCTGGAGTTCACGCAGTTGCAGAAAGTGAAGGAGAAGGAGTGCGATGCCTTAATACAGAACAGTTCTAATGCAAGAATTGCGGCCACACCTGTTGTCGGTTGGCCTCCGATCCGTTCTTTCAGAAAGAATCTTGCTGGAACTCCTAAACTCTCCACCGAATCCCCGAACCAGAACTCTAAGGCTGCAAAAAAGCCTGAGAATGAGAGGAAGAGCCTCTTTGTGAAAATTAACATGGATGGCATTCCAATCGGAAGAAAAGTAGACCTCAAGGCCTACGATAGCTACGACAAACTATCATTAGCTGTTGATGAGCTCTTCGGAGGCCTCATGACAGCACAAATGGATCCACTTGCTCTTGGTACACCAAAGATCTCCGAAGAAAAACACGCGATTACTGGCTTATTAGATGGTTCAGGTGAATTCACTCTGGCTTACGAAGACGATGAAGGGGATCGGATGTTGGTTGGTGATGTCCCTTGGGACATGTTTGTTTCCACTGCCAAGAGATTGCGTGTTTTAAAGAGCTCCGATCCATCTGCATTGTCTCTCGGAGCAGTGAGCAGGAAAAGATCAAATGGATCTTTGATGTGCAACTTCAATGTTGTGGCATAG

**Protein sequence**

MAEEKKPLDWSTAEERKLELRLGLPGEGEDWSPSQEKGKKHLPLETSLSLGHVSKVPRSNNFTASSATKREVVAAFQQPKQLEFTQLQKVKEKECDALIQNSSNARIAATPVVGWPPIRSFRKNLAGTPKLSTESPNQNSKAAKKPENERKSLFVKINMDGIPIGRKVDLKAYDSYDKLSLAVDELFGGLMTAQMDPLALGTPKISEEKHAITGLLDGSGEFTLAYEDDEGDRMLVGDVPWDMFVSTAKRLRVLKSSDPSALSLGAVSRKRSNGSLMCNFNVVA

**HcIAA5 >evm.model.scaf_1096.12**

ATGGAAGGGGATCGCCGTTACGGCGGAGGCGCTGCGTCGCCTGCGTCGGTTTCCTCTGTACCGAAGGCGGCGGAGCGGGATGTGGCGGTGGAGGATGAAGTCTCGGCGGGAGTGGCCGAGGGGGAGGAAGGAGAGAGTGATCGAGATGACGAGGGATTGGAGCTAGGGTTGACGCTGGGTGCGGCGAGGAAGTGGAACCCTCCGCCGGTGCCGTGGAGTCCTTTCTTACGTATCCTGACGGCGAAGGACCTCCCCTCCCTGGCGTCGCTTGCTCCTCCGAGTCCCCCTTCCGCCTCCTCCGTCTCGTCGTCCTCCGTCACCAACCTGAGAGGCGGAGGAACTGGGAGAAAGCGATCGGACGAGTCGGCTTCCCCGGATGTCGGTGGGTCTCCTCAGCCTCCCAGTCAGATGGTGGTGGGGTGGCCACCTATCAGGGCATTTAGGATGAACAGCTTGTTCAACCAATCCAAAGAGCATACACCTGAGACTACTTCTGTAGCTATCAAGAAAAGTGTCAGCAAGGACCAGAGCTGTAAGGATGAAGGAGGCAGAAGAACGGAATTGAGAAATTCAATGTTTGTTAAAGTTAAAATGGATGGTGATCCTATTGGTAGGAAGGTGGATCTCAATGCCCACCACTCTTATGAAGCTCTTGCAGTTTCTCTTGAGCTCATGTTTCAGAAACCCACCATGGCTACCAACCTTCTGGTCTCATTTGATGGAGCAAAGGCTTTGAAATTATCGGATAGTTCATCTGAGTTCGCTCTTACCTACGAAGACAAAGACGGGGACTGGATGCTGGTCGGAGATGTTCCATGGGGAATGTTTGTGGAAACTGCCAAGAGACTTAGAATCATGAAGATCTCTGATGCAACTGGTCTTAGTATATGA

**Protein sequence**

MEGDRRYGGGAASPASVSSVPKAAERDVAVEDEVSAGVAEGEEGESDRDDEGLELGLTLGAARKWNPPPVPWSPFLRILTAKDLPSLASLAPPSPPSASSVSSSSVTNLRGGGTGRKRSDESASPDVGGSPQPPSQMVVGWPPIRAFRMNSLFNQSKEHTPETTSVAIKKSVSKDQSCKDEGGRRTELRNSMFVKVKMDGDPIGRKVDLNAHHSYEALAVSLELMFQKPTMATNLLVSFDGAKALKLSDSSSEFALTYEDKDGDWMLVGDVPWGMFVETAKRLRIMKISDATGLSI

**HcIAA6 >evm.model.scaf_506.60**

ATGGAGACTGCCTTGGGTACTAACAACCTTAGGGATACCGAACTAAGGTTGGGTCTACCGGGCATCGATGTGGTTGGGTCAGAGAAATTGAGGGCCACGAAAGGTAGTAAACGGGCACTTTCGGAGGATGGAACTCAGAGTTGTGCTAAAAGCAAGTCCTTCGAAGAGGTCTATGACGATCTAAGTGGCCTAGAAACTCCAATGACAAAGGCACAAGTAATTGGATGGCCACCGATCCGATCCTACCGGAAGAACTTACTATTAGTTCAGGTTCAAGCCGCGGCGAATGCAACAGGATTGTATGTGAAAGTAAGCATGGATGGAGCTCCTTATTTGAGAAAGATCGATCTCAAGGTGTACAAAGGATATAAGGAGCTTAGAGAAGCATTGGACAAAATGTTCAAATGCTTTTCTTTAGGAGAGGTGCAAGGGAAGGAAGGAGGCAGTGGATGTGAGTATGCTATCACTTACGAGGATAAAGATGGAGATTTAATGTTGGTTGGTGATGTTCCATGGGAGATGTTCATCTCTTCTTGCAAAAGGCTGAGAATAATGAAAGGGTATGAAGCGAGAGGATTACAGTCTAAACATTGA

**Protein sequence**

METALGTNNLRDTELRLGLPGIDVVGSEKLRATKGSKRALSEDGTQSCAKSKSFEEVYDDLSGLETPMTKAQVIGWPPIRSYRKNLLLVQVQAAANATGLYVKVSMDGAPYLRKIDLKVYKGYKELREALDKMFKCFSLGEVQGKEGGSGCEYAITYEDKDGDLMLVGDVPWEMFISSCKRLRIMKGYEARGLQSKH

**HcIAA7 >evm.model.scaf_717.7**

ATGGAAGCAGAGACGGACAACCTCAAGGCCACCGAGCTCAGGCTTGGCCTCCCCGGCAGCGGCGCCTCCGACGCTGCACCACCAGCGTCCTCGAGGCCATCCAAGCGCTCCCTCAACGACCAGAGTCAAGCCCAGAAAGCGCAGCTGGTCGGGTGGCCGCCCGTTCGGTCCTACCGGAAGAACGCAATGAAGGCCGAGGCCGGGCTGCTCGTCAAGGTCAGCATGGACGGCGCTCCCTACTTGAGAAAGATCGACTTGACGGCGTTCAAAGGCTACAAGGAGCTGAGCAAGGCCTTGGAGGACATGTTCAAGGCCTTCGAGGGTTGCAAAGGCTCAGATTACGCCATCGCCTACGAAGACAAAGATGGGGACTTGATGCTCGCCGGAGATGTTCCTTGGGAGATGTTCACATCTTCTTGCAAGAAGTTGAGGATCATAAGAGGAGGGTCTGAGTCCACCAAGAAGTGA

**Protein sequence**

MEAETDNLKATELRLGLPGSGASDAAPPASSRPSKRSLNDQSQAQKAQLVGWPPVRSYRKNAMKAEAGLLVKVSMDGAPYLRKIDLTAFKGYKELSKALEDMFKAFEGCKGSDYAIAYEDKDGDLMLAGDVPWEMFTSSCKKLRIIRGGSESTKK

**HcIAA8 >evm.model.scaf_38.101**

ATGCCGCCGCCGCCGCCGCCTGTGGAGCGGGAGTTTTCCGTTGGAGGCGGCGAGGAAAGGGCTCTCAATCTCAGGGCGACGGAGCTCAGGTTGGGGCTGCCTGGGTCAGAGTCGCACCACCAGGACGACGAGGTCGGGCTAACCCTAGATCTTCTCCCCAAGAGCTTCGCCTCCAGCGCCAAGAGGGGGTTCTTCGACGCCATCGATAATGCCGGGAAGTGGGGCTTTGCGGCCGGCGAGGGCGGGTCTGAGGTGGACTTGGTGAAAGGCGGCGGTTTGTTATCGACTTCGGGCCAGGCCTCTGAACAAGGGAATGTAGGGAAGGTGGCGGCTCACTGCGGCGGTTCCATTGGGAGAGATCACTCGGTGGCTCCGGCAGCGAAGGCACAGGTGGTTGGTTGGCCACCAATCCGTAGTTATCGAAAGAATACCATGGCCACAAATCCATCGAAGACTAAAGATGATGCTGAGGGTAAACAATTGCTAAATTGCCTATATGTGAAGGTTAGCATGGATGGAGCTCCTTATCTTAGAAAAGTTGATCTCAAAATATACAAAAATTACAAAGAACTTTCATCGGCACTCGAGAAAATGTTCAGATGCTTCACGTTCGGTCAGTGTGATTCTCAAGTAACACCAGGCAATGAGGGATTATCCGAGAACCGTTTGACCGACCTTCTAAATGGTTCTGAATACGTACTTACTTACGAAGACAAGGATGGAGACTGGATGCTTGTTGGTGATGTGCCGTGGGGGATGTTCACAGACTCTTGTAGGCGGCTTCGGATCATGAAAAGTTCAAATGCTATTTGTCTAGCTCCGAAGGGCATGAAGTCCCGGAACCACAACTAG

**Protein sequence**

MPPPPPPVEREFSVGGGEERALNLRATELRLGLPGSESHHQDDEVGLTLDLLPKSFASSAKRGFFDAIDNAGKWGFAAGEGGSEVDLVKGGGLLSTSGQASEQGNVGKVAAHCGGSIGRDHSVAPAAKAQVVGWPPIRSYRKNTMATNPSKTKDDAEGKQLLNCLYVKVSMDGAPYLRKVDLKIYKNYKELSSALEKMFRCFTFGQCDSQVTPGNEGLSENRLTDLLNGSEYVLTYEDKDGDWMLVGDVPWGMFTDSCRRLRIMKSSNAICLAPKGMKSRNHN

**HcIAA9 >evm.model.scaf_899.17**

ATGTCACCGCCTCTCGAGCATGACTACATAGGCCTCTCCGAGCTCCACGCTCCGGCCGCCGAGGATGGAACCCTCAACCTGAAGGAGACGGAGCTGAGGCTCGGCCTCCCGGGGTCCGGGTCCCACGACCGGAGTGACTTGGCGGGGGGGCTCACTTTGGGCCTCATCCCCAAGCCCTTCGTCTCCGGCGCGAAGCGAGGGTTCTCGGACGCCATCGACGGGGCCGGCAAGTGGGGACTCGCGGCCTTAGGGGGTAGATCTGAGGTGGATTCTGGAAAATGTGGCGCCTTGTTCTCCCCTAAGGGGGAGAACGGCGACGGGCAGTTGCCCGCTCTCGGAAATGCCGGAAAAGGCATGGACGCGGTGGCCAAGGCGACCGAGCACGAGAGAAAGGCCGCCGGACTGGTCGGGAATTTGACTGGGAATGAGCGTGGAGTCGCGCCTGCGGCAAAGGCACAGGTGGTAGGTTGGCCGCCAATTCGTAGTTATCGTAAGAACAACATGGCTACAAATCCATCAAAGAACAAAGAAGATGCTGATGGGAAGCAAGGATCAGGATGCCTTTATGTTAAAGTCAGCATGGATGGAGCACCATACCTTAGGAAAGTTGATCTGAACATATACAAGAACTATAAGGAGTTCTCATTGGCACTAGAGAAAATGTTTAGTGGCTTCACTATGGGCCAGTGTGATTCTCATGGATTACCAGGCCGAGATGGATTGACTGACTGCAAGTTGACTGATCTTCTAAGTGGATCAGAATATGTGCTCACATATGAGGACAAAGATGGTGATTGGATGCTTGTTGGGGATGTGCCATGGGAGATGTTCACTGACTCTTGCAAGAGGCTGAGGATAATGAAGGGTTCAGATGCAATTGGACTTGCTCCAAGGGCCATGGAGAAATGCAAGAACCGTAACTAG

**Protein sequence**

MSPPLEHDYIGLSELHAPAAEDGTLNLKETELRLGLPGSGSHDRSDLAGGLTLGLIPKPFVSGAKRGFSDAIDGAGKWGLAALGGRSEVDSGKCGALFSPKGENGDGQLPALGNAGKGMDAVAKATEHERKAAGLVGNLTGNERGVAPAAKAQVVGWPPIRSYRKNNMATNPSKNKEDADGKQGSGCLYVKVSMDGAPYLRKVDLNIYKNYKEFSLALEKMFSGFTMGQCDSHGLPGRDGLTDCKLTDLLSGSEYVLTYEDKDGDWMLVGDVPWEMFTDSCKRLRIMKGSDAIGLAPRAMEKCKNRN

**HcIAA10 >evm.model.scaf_357.107**

ATGTCACCGCCTCTCGAGCATGACTACATAGGCCTATCCGAGCTCCACTCCCCGGCCGCCGTCGCCGTCAGCGCAGCCGAGGTTGGGGCCCTCAACCTGAAGGAGACGGAGCTAAGGCTCGGCCTCCCGGGGTCCGAGTCGCCTGACCGGAATGACTTGGCGGGCGGCCTCAGCTTGGGCCTCCTCCCCAAGCCCTTGGTATCCGGCGCAAAGCGAGGGTTCTCCGACGCCATCGACGGGGCCGGCAAGTGGGGACTCGCCGCCGTTGGAGGTAGATCTGAGGCGGATGCTGGTAAAGTTGGCGCCTTGTTCTCCTCCAAAGGGGAGAACTGCGACGGGCAACTGCCTGCCCTCGGGAATGCGGGAAAAGGCAAGGACGCGGTCGTGAAGGCGACCGAGAATGAGAGGAAGGCTGCCGGACTGGTAGGGATTGCGAATGGGAATGAGCGTGGCGTCGCGCCTGCTGCAAAGGCGCAGGTAGTAGGTTGGCCACCAATTCGTAATTATCGTAAGAACAGTATGGCTACAAATCCACCAAAGAACAAAGAAGATGCTGATGGAAAGCAAGGATCAGGGTGTCTTTATGTTAAAGTCAGCATGGATGGAGCACCATACCTTAGGAAAGTTGATCTGAAGATGTACAAAAATTATAAGGAGTTCTCGTTGGCACTAGAGGAAATGTTTAGTGGCTTCACCATTGGCCAGTGCGATTCTCATGGAATACCAGGTTTTTGCGATGGATTAGCTGACAGCAAGTTGACTGATCTTCTGAGTGGATCAGAATATGTGCTCACTTATGAGGACAAAGATGGTGATTGGATGCTTGTCGGGGATGTGCCATGGGAGATGTTCACCGACTCTTGCAGGAGGCTGAGGATAATGAAGGGTTCAGATGCGATTGGACTTGCTCCAAGGGCCATGGAGAAATGCAAGAACCATAACTAG

**Protein sequence**

MSPPLEHDYIGLSELHSPAAVAVSAAEVGALNLKETELRLGLPGSESPDRNDLAGGLSLGLLPKPLVSGAKRGFSDAIDGAGKWGLAAVGGRSEADAGKVGALFSSKGENCDGQLPALGNAGKGKDAVVKATENERKAAGLVGIANGNERGVAPAAKAQVVGWPPIRNYRKNSMATNPPKNKEDADGKQGSGCLYVKVSMDGAPYLRKVDLKMYKNYKEFSLALEEMFSGFTIGQCDSHGIPGFCDGLADSKLTDLLSGSEYVLTYEDKDGDWMLVGDVPWEMFTDSCRRLRIMKGSDAIGLAPRAMEKCKNHN

**HcIAA11 >evm.model.scaf_189.134**

ATGGACGAAGATTTCAAGAACAGAGGGCTTCCGCAACTGCTTCCGTACGAGCAAGATTTGGAACTGAGGCTCGGGCTTCCCGGAGGAGAAGACGAGCCTAGAGCTATTTCAACTGGCAAAGGTCTCTTCTCCATGGCCTGCCTTTTCTTCTTCCCATCTAAAAATGTGTCCGTCACTTCGCATCATCTTCCTGTCTCTTTAGGTCCGAAGCTGAAGCAGCAAGTTGGATTCCTTCAACTGCAGAGCATGGAGGTGAAAGATGAAGCACATTTCGCATCTGGAAGCGCATATGGTTCCTTGACAAGGATCACCACTGTACTAGTCGTTGGCTGGCCCCCTCTGAGTTCCTTCAGAAAGAACCTAGCGAGGAGCAGCTCACATAGGCCGCTCATCGAGTCTGAAAACGAAGATGTGGAGGAACCGGGCAACAGCAAGAAGAATTTGCTTGTTAAGATCAACATGGACGGCGTCCCGATTGGAAGGAAAGTAGACTTATACGTCCATGATAGCTATGAGAAGCTCTCTTTGGCCGTGGAAGAGCTATTTGCAGGCCTTCTTGCAGGTAATCCCCACAGTGAAGCAGCAAAACAAATGTTTAGGGGCTTGTTAAATGGTACAGGCGAATACACTTTGGTTTATGAAGACAACCAAGGTGACAGTATGCTTGTTGGTGATGCTCCTTGGAACATGTTTACTTCAGCAATTAAAAGGCTGAGGGTCATGAAGACTTCTGATATTTCTGCACTGAAATTTACTTTAAGTCCATTTGGCGATTAA

**Protein sequence**

MDEDFKNRGLPQLLPYEQDLELRLGLPGGEDEPRAISTGKGLFSMACLFFFPSKNVSVTSHHLPVSLGPKLKQQVGFLQLQSMEVKDEAHFASGSAYGSLTRITTVLVVGWPPLSSFRKNLARSSSHRPLIESENEDVEEPGNSKKNLLVKINMDGVPIGRKVDLYVHDSYEKLSLAVEELFAGLLAGNPHSEAAKQMFRGLLNGTGEYTLVYEDNQGDSMLVGDAPWNMFTSAIKRLRVMKTSDISALKFTLSPFGD

**HcIAA12 >evm.model.scaf_189.121**

ATGACGCCGCCAATGGAGCACGACTACATGGGCGTCGACGACACCGAGCTCCGGCTCGGCCTGCCCGGCTCCAACGGCCGGGTGGTGGTCGACGGAGGCGCAACCGCCGGCCTCACACTGGGCGGCTCGAAAAGGGGTTTCTCTGACGTCATACACCGGGCTTCCTTCTCTGAGGCAGCAGGGGAGGACGCCGGTGGAGCAACCGAAGCGGCGCCGCGACCGCCTCCGGCGAAGACACAGATTGTTGGTTGGCCACCGATCCGTAGTTACCGAAAGAATACAATCGCATCAAATAGTTCGAAGAACAATGATGATGCCGATGCGAAGCAAGGGAATGAATGCCACTACGTGAAGGTCAGCATGGATGGAGCTCCCTACCTGAGAAAAGTTAACCTCAATATGTACTCCGACTACAAGAAGCTGTCACTAGCACTAGAGAAGATGTTTACTTGCTTCACCTTCGGCCAGTGTGGTTCATATGGAGTGAGTGCTAGAGAGATGACTACTGAGGGCAGAGTGTTGGACCTTCTTCAAGCATCTGAACATACCCTTACCTATGAGGACAAGGATGGGGATTGGATGCTCGTCGGCTGA

**Protein sequence**

MTPPMEHDYMGVDDTELRLGLPGSNGRVVVDGGATAGLTLGGSKRGFSDVIHRASFSEAAGEDAGGATEAAPRPPPAKTQIVGWPPIRSYRKNTIASNSSKNNDDADAKQGNECHYVKVSMDGAPYLRKVNLNMYSDYKKLSLALEKMFTCFTFGQCGSYGVSAREMTTEGRVLDLLQASEHTLTYEDKDGDWMLVG

**HcIAA13 >evm.model.scaf_107.22**

ATGGCGGAGAAGTATAAACGTGGAGAATGGAACACCTACGCTGCCGCTGTGGACGAAAAGCTAGAACTAAGGCTTGGCCTCCCTGGAGGTGAACACTGGTCGGCGGTGCTGGAGAAGAGAGAGCTTCCTATTGATCCTTCTCTCTCTCTTGGACACATCTCCAAGCTTCCTAAGAGAAACAACGTTACTGCTTCCTCTGCAACCAAAAGAGGCTTAACGAGTCCAGTTGTTTCTAAAACAGAAGCTTTTCGCCAGCACAAACGGCTTGGATTCCTCCAATCGCAGGCCAAAGGAACAGATTCATTTCAGAAAACAAGTGGAAACACAGAGCAGCAGCAGAGCCTCGTGGGGAAATCCAAGAATTCCACTATAGTGCAAATCAATTCTGATTCAAGCAGAATCATGCCAGCACCTGTTGTTGGTTGGCCTCCCATCCGCTCATTCAGAAAGAATCTTGCTGGAGCTGCTAAACTGTCGATTGAGTTTCAAAATGAAAACTCCAAAGTTCTAAAGAAGTGCGAGAATGAGAAGAAATGCCTGTTTATGAAAATTAACATGGATGGCATACCCATTGGAAGAAAAGTAGACTTGTTGGCATATGATAGCTACGATAAGCTCTCAATGGCTGTCGATGAGCTCTTCCGAGACCTCATGACAGGTACAAATTTACTGTGCAATCATGTTACTGCAGAAAAGCTTGTTATTACTGGCTTATTAGATGGCAGTGGGGAATACACTCTGGTTTACGAAGACGATGAAGGGGACGAGATGTTGGTTGGTGATGTTCCATGGGAAATGTTTGTTTCCTCGGCGAAGAGACTACGAGTTTTGAAGAGCTGTGATCTCTCTGCATCATCAGTAAATGTGCTTGTTCCAGGAACATTGAGAAAGGAAAGAGCAAATGTGCCTTGA

**Protein sequence**

MAEKYKRGEWNTYAAAVDEKLELRLGLPGGEHWSAVLEKRELPIDPSLSLGHISKLPKRNNVTASSATKRGLTSPVVSKTEAFRQHKRLGFLQSQAKGTDSFQKTSGNTEQQQSLVGKSKNSTIVQINSDSSRIMPAPVVGWPPIRSFRKNLAGAAKLSIEFQNENSKVLKKCENEKKCLFMKINMDGIPIGRKVDLLAYDSYDKLSMAVDELFRDLMTGTNLLCNHVTAEKLVITGLLDGSGEYTLVYEDDEGDEMLVGDVPWEMFVSSAKRLRVLKSCDLSASSVNVLVPGTLRKERANVP

**HcIAA14 >evm.model.scaf_285.12**

ATGGCGAGCGGATTGGGCCTGGAGGCGACCGAGCTGCGCCTGGGGTTGCCCGGAGCCGGAGGGGAAGGCGAGGGCGCGAAGAAGAGGGGATACGAGGAGACAATCGACCTGAAGCTTCAGCTCCAGACACCCGCTGATGTGAAGGAACCGGAGAAGGGCGCCGATAATATGGTCAAGAAAACTCCGAGCGTCAAGAACGTTGCTCAATGCGGCAGCGTTCTCGAACCGGAAAAACCACCTGCTTCCAAGGCACAGGTTGTGGGTTGGCCACCGGTTAGATCATTTAGGAAGAACATCCTTTCTTTCCACTCTGACAAGGGGGGCAGCAACATGAAGGAGGAGGGTGAAAAGTCTGGCAACTCGATGGCCTCCTTCATGAAGGTGAGCATGGATGGTGCGCCATACCTGCGTACGGTGGATCTAAAGATGTACAAGAGCTATCATGAGCTCTCCATGGCCTTGCAGAAGATGTTCGGCTCCTTCACCAACAATGGAAACTGTGGCTCGCAGGGAATGAGTGGTAAGGACTTCATAAACGAGAGCAAGGTGATGAATCTGCTGAATGGATCCGAATATGTCCCAACCTATGAAGACAAGGATGGAGATTGGATGCTTGTCGGTGATGTTCCATGGGAGATGTTTGTCGACTCATGCAAGCGTTTGCGAATCATGAAGGGATCGGAAGCCAATGGACTCGCACCAAGAGCTCTGGAGAAGTGCAAGAACTCGAGCTGA

**Protein sequence**

MASGLGLEATELRLGLPGAGGEGEGAKKRGYEETIDLKLQLQTPADVKEPEKGADNMVKKTPSVKNVAQCGSVLEPEKPPASKAQVVGWPPVRSFRKNILSFHSDKGGSNMKEEGEKSGNSMASFMKVSMDGAPYLRTVDLKMYKSYHELSMALQKMFGSFTNNGNCGSQGMSGKDFINESKVMNLLNGSEYVPTYEDKDGDWMLVGDVPWEMFVDSCKRLRIMKGSEANGLAPRALEKC

**HcIAA15 >evm.model.scaf_440.26**

ATGACAAGCGGGTTACGGATGGAGGAGACTGAGCTGCGTCTGGGACTGCCGGGCGGCGACGCTGGAGAATTAGCAGAAGTGACAGTGAGGAGCTCCGGGAAGAGAGGCTTCCTGGAGACAATTGATCTGAAGCTGAAGCTTCAAACCCCGGCGGAGAGCAAGGAATTAGAGTCCTCCGAGCAAGGAAACAAGGCCACTGATCATCACGAGAAGTCATCTGCTCCCAAGGCACAGGTGGTGGGTTGGCCTCCAGTTCGATCATTCAGGAAGAACATTCTCAACGTGCAACAACAGTCTGAGAGCAAGAACAAGGTAGAACAAAGCGAGAAGACCACCGCCGCCGCCGCCGCCGCCTTTGTGAAGGTGAGCATGGACGGGGCTCCTTACCTTCGTAAGGTCGATCTCAAGACCTACAAGAGCTATAAGGACCTCTCCACGACATTGCAGAAGATGTTCTCCTCCTTCGCCACCGCCGGAAATTATGGGTCTGAACAAGGAGTAAGCGTGAGGGATTTCATGAACGGCGCCGGCAAGGTGACGACGGCGCTGGTGATCAATGGATCCGAGTATGTGCCAACTTATGAAGACAAGGATGGCGACTGGATGCTCGTAGGTGACGTTCCTTGGGAGATGTTTGTTGACTCCTGCAAACGCTTGCGAGTAATGAAGGGATCAGAGGTCGTCGGACTTGAGCCGAGGCCAATGGATAAACGCAAGAACAGAGGCTGA

**Protein sequence**

MTSGLRMEETELRLGLPGGDAGELAEVTVRSSGKRGFLETIDLKLKLQTPAESKELESSEQGNKATDHHEKSSAPKAQVVGWPPVRSFRKNILNVQQQSESKNKVEQSEKTTAAAAAAFVKVSMDGAPYLRKVDLKTYKSYKDLSTTLQKMFSSFATAGNYGSEQGVSVRDFMNGAGKVTTALVINGSEYVPTYEDKDGDWMLVGDVPWEMFVDSCKRLRVMKGSEVVGLEPRPMDKRKNRG

**HcIAA16 >evm.model.scaf_47.162**

ATGGCGAGCTACGTCGAGGATACCGAGCTGCGCCTCGGTTTGCCGGGATCCGATGCGCCGGCGGCAAGGGGTAGCAAGCGATCGCTTCCGGAGGATAGCGCAGCGGGGGACGAATCCTGCTGCCGGAATGACGGATCTGCGTCGGCGGCGAAGGCGCAAGTGGTTGGGTGGCCGCCGATCGGAACGTACAGGAAGAAGCTGACGATGACAGTGGAGCCGGAGGTTTCTGGATTGTATGTGAAGGTTAGCATGGATGGAGCTCCTTACTTGAGAAAGATTGATCTCAAGATTTACAAAGGGTACCAGGAGCTCAAAGAGGCCTTGGACAGCATGTTCAATTGCTTTTCCCAAGGAGAATTATCAAGGAAAGAAGGGTGCAATGGATCTGAATATGCTATTGCTTACCAAGACAAAGATGGAGATCTGATGCTGGTTGGAGATGTTCCTTGGGAGATGTTTACATCTTCTTGCAAAAGGCTGAGGATAATGAAAGGATCTGAAGCAAGAGGTTTAGAATTTAGACAATAG

**Protein sequence**

MASYVEDTELRLGLPGSDAPAARGSKRSLPEDSAAGDESCCRNDGSASAAKAQVVGWPPIGTYRKKLTMTVEPEVSGLYVKVSMDGAPYLRKIDLKIYKGYQELKEALDSMFNCFSQGELSRKEGCNGSEYAIAYQDKDGDLMLVGDVPWEMFTSSCKRLRIMKGSEARGLEFRQ

**HcIAA17 >evm.model.scaf_1004.1**

ATGGAGTTGGAGCTCGGCCTTTCACTCCCCAGCTCAGATATGATGATGGAGAAGAGGATCATGACTAACTTTTGCAAGAAAAGAAGCTTCGACGAGGTGCTCGACGAAAAGACTGTCACTTTACCCCTCTTCGTGCAGAGGGAGGAAGACGGCGACGGCTACAGTGACAGAGGATTCGAGCAGTCCGAAGAGCTTAACTATGGTGGAGGTAGAGTTATAGTTGGTTGGCCGCCGGTGAAATTGCCGGGCAAGAGGACGAACTGCGTGAAGGTGAACATGGAGGGGATTGCGATAGGAAGGAAGGTGGACCTCTCCCTTCATGATTCTTACCAGGCTCTCTTCCGCACGTTGTCCATGATGTTTCCTAAGAAACATGGAGACTTGGCATATGCAAATGTACGTCGTACAGAGGAAGAGGATGGAGAATATAACTATAAAGTGACTTATGAGGATGAGGATGGGGACTGGATGCTGGTCGGAGATGTGCCGTGGGAAGCTTTCATCATGTCAGCGAAGCGTCTCAGGATAATCAACTAG

**Protein sequence**

MELELGLSLPSSDMMMEKRIMTNFCKKRSFDEVLDEKTVTLPLFVQREEDGDGYSDRGFEQSEELNYGGGRVIVGWPPVKLPGKRTNCVKVNMEGIAIGRKVDLSLHDSYQALFRTLSMMFPKKHGDLAYANVRRTEEEDGEYNYKVTYEDEDGDWMLVGDVPWEAFIMSAKRLRIIN

**HcIAA18 >evm.model.scaf_412.59.2**

ATGGAAGATGACCATGAGAGAATGGGAGATGCATGCCCTCAGCTGCTAAACTTAATCCCAAATGAAAAAGTCAGGAAAAAAAGACTTGGGAGTTTAGATGTCTCAGAAGAGAAGAAATTGGAGCTGACGCTTGGGCTTCCTGGAGGAGTTGGGGAAGAAGAGGAGCCCTCTGTTCTATCTTCTTGTATCTTTTCAAAGGTTTCCAAGACTTCTTCCAGAAGGAGAGTTTTAGGTTCTGAGCAGCAAGAAGCCGGATTGTTCCAGTCGCAGAGCAGAGGTGGGTATGGAACGGAATGGCCGCAGAAGACAACTTGGAACTGTGAGAAATTTGATGAGCAGCAAGGCCTTGAGAAGGAGAAAGTACATGGTGGCACTGAGGCTGAAGCTGGATCAAACACCAGCTTCCAGACCAGAGCTTCTGCCGGGGTTGCTGTTGTTGGTTGGCCTCCGATACGAGCCTTCAGAAAGAACCTAGCAAGTAGCTCAGTTAAGCAATCTGTGGAGCTTCAGAATGGAAATATAGATGAAAAAGTGAAGGTTGCCAGCTGCAAGAAGGGTTTGCTCATCAAGATTAACATGGATGGTATCCCTATCGGAAGAAAGGTGGATCTGAATGCCTTTGGTAGTTACAAGAAGCTTTCATTTGCGGTTGAATATCTCTTTCTAGACCTTCTTGAAGCCCAAAAGGAGACTTCTTCAAATGACCTTCGAATGAATGAGGAAGCAAAACAAGCATTCAAAAATTTATTAGATGGCTCTGGCGAATACACTTTGGTTTATGAAGACAATGAAGGTGACAGAATGCTTGTGGGGGATGTTCCTTGGGACATGTTTGTTTCTTCAGTAAAAAGATTGAGGGTGTTAAAAAGTTCTGATCTTTCCTCTCTACATTTAGGATTGGGAAGCCAGAAAAGAACAGCAACTGAGTGTTGA

**Protein sequence**

MEDDHERMGDACPQLLNLIPNEKVRKKRLGSLDVSEEKKLELTLGLPGGVGEEEEPSVLSSCIFSKVSKTSSRRRVLGSEQQEAGLFQSQSRGGYGTEWPQKTTWNCEKFDEQQGLEKEKVHGGTEAEAGSNTSFQTRASAGVAVVGWPPIRAFRKNLASSSVKQSVELQNGNIDEKVKVASCKKGLLIKINMDGIPIGRKVDLNAFGSYKKLSFAVEYLFLDLLEAQKETSSNDLRMNEEAKQAFKNLLDGSGEYTLVYEDNEGDRMLVGDVPWDMFVSSVKRLRVLKSSDLSSLHLGLGSQKRTATEC

**HcIAA19 >evm.model.scaf_269.7**

ATGGCAAGGGTTTGCGGGTTCGACGGAGGCGTGGCGTCGCCGGAATCGATGTCTTCCATGTCGAAGGCGGTGGCTAGGGAGGCGGATGATTTGGAGGTGGAGGATGTGGGTGGTGGGTCGGTGGGGGAGAGGGAGGAGGAGGTGATGGATGAAGAGTGGGGTGACAAGGCGGAGGCAGAGCTAGAGCTGGGGCTGGCTTTAGGCTCGGTAAAGAAGGAGAAGGCGGCAGCCTCTGCGCCGCACCGGGCATCGTGCTGCCGCATCCTGACAGCGAAGGATTTCCCCTCGCGTGCTTCGCGGAGGTCTCCCTGCTCCTTTGTCTCAGCATCATCCGGCACCAACTTGGGCGGAGGGGCCGGCGTCGTCGGGACCAAGCGTACCGCAGAATCTGTATCCCCGGACGTCGGCGTTTCCCCACATCCTCCTAGTCAAGCGTTGGTTGGATGGCCGCCTATAAGGGCTTTTAGGATGAACAACTTGTCCAACCACTCCAAAGACACCGCTTCCAACGTCGAAGCCACCACTGTTAAGAAGACCAACCGCAACAACGGTACCAACAGCACCAGTCTGGTGGTCAATGGTTGCAAGGATCAGGAGAACAAAGGAAAGGTGTTGCGGGGTTCTTTCTTCGTTAAAGTGAAAATGGACGGTGACCCCATTGGAAGGAAGGTGGATCTTCATGTCCATAACTCATATGAAACTCTTTCTGCTGCACTTGAGCTCATGTTTAACAAGCCAACCATGACATTAACCCTCACTAACACTGTCCATGGATCAAGGTTCTCAAAGTTATTAGATGGTTCTTCTGAGTTTGCTCTTACTTATGAAGACAAGGATGGTGATTGGATGCTTGTTGGAGATGTGCCTTGGCAAATGTTTCTGGAAACAGTTAAGAGACTTCGAATCATGAAAACCTCAGATGCGCATGGGCTAAGCAAATCAGCGACCAGATTGCTTTTGTTCAACAAGGCCAGTAGATCACTAGTTGCCTGGAGAGGAAGTTGA

**Protein sequence**

MARVCGFDGGVASPESMSSMSKAVAREADDLEVEDVGGGSVGEREEEVMDEEWGDKAEAELELGLALGSVKKEKAAASAPHRASCCRILTAKDFPSRASRRSPCSFVSASSGTNLGGGAGVVGTKRTAESVSPDVGVSPHPPSQALVGWPPIRAFRMNNLSNHSKDTASNVEATTVKKTNRNNGTNSTSLVVNGCKDQENKGKVLRGSFFVKVKMDGDPIGRKVDLHVHNSYETLSAALELMFNKPTMTLTLTNTVHGSRFSKLLDGSSEFALTYEDKDGDWMLVGDVPWQMFLETVKRLRIMKTSDAHGLSKSATRLLLFNKASRSLVAWRGS

**HcIAA20 >evm.model.scaf_41.21**

ATGTCACCGACTCTCGAGCATGACTACATAGGCCTTTCGGAACTCCAGCCCTCCGCGGCGGTAGCCACCGCGGGGGATGGGGTCCTCAATTTGAAGGAGACGGAGCTGAGGTTGGGTCCGCCAGGGTCCGAGTCCCCCGATCGGAAGGACTTTGTCGGGGGTCTCACCACCCCGGGCCTCTTCTCCAAGCCATTCGTATCCGGCGCCAAGCGAGGGTTCTCCGACGCCATGGACGGGGCCGGGAAGTGGGGACTTATCGCGGGCGGGACCGGATTCGAGGTGGATGCTGGAAAAGGATGCGCCTTCTTCTCCTCCAGGGGGGATAACGGAGGCGGAAAGCTGTCCGGACTGGGGAATGCGGACAAGGACACGGTGTCGAAGGTGGTGGCGCTGGAGAGGAAGGACGCCATCCAGGTTGAGAATTCCGCTGGAAGTGAGCGTGGCGTCGCCCCCGCTGCAAAGGCACTGGTTGTAGGTTGGCCACCAATACGTAGTTACCGAAAGAACAGTATGGCTACAAATCCATCAAAGAACAAAGAAGATACCGAAGGAAAACAAGGCTTGGGATGCCTTTACGTGAAGGTTAGCATGGATGGAGCACCATACCTTAGGAAAGTTGATCTGAACTTGTATAGCACCTATAAGGAATTTTCATCAGCACTTAAGCAGATGTTTAGTGGCTTCACCATCGGCCAATATGATTCTCATGGAGTACCAGGACAAGATGGATTAACTGAGAGTAAGTTGACTGATATTTTGAGTGAATCGGAATATGTCCTCACATATGAGGACAAAGATGGTGATTGGATGCTTGTTGGGGATGTGCCATGGGAGATGTTCTCGGGCTCTTGCAGAAGGTTGAGGATAATGAAGGGTTCAGATGCAATTGGACTCGCTCCAAGGCCATGGAGAAATGAAAGAACTATAACTAAGGCCAAATGA

**Protein sequence**

MSPTLEHDYIGLSELQPSAAVATAGDGVLNLKETELRLGPPGSESPDRKDFVGGLTTPGLFSKPFVSGAKRGFSDAMDGAGKWGLIAGGTGFEVDAGKGCAFFSSRGDNGGGKLSGLGNADKDTVSKVVALERKDAIQVENSAGSERGVAPAAKALVVGWPPIRSYRKNSMATNPSKNKEDTEGKQGLGCLYVKVSMDGAPYLRKVDLNLYSTYKEFSSALKQMFSGFTIGQYDSHGVPGQDGLTESKLTDILSESEYVLTYEDKDGDWMLVGDVPWEMFSGSCRRLRIMKGSDAIGLAPRPWRNERTITKAK

**HcIAA21 >evm.model.scaf_614.34**

ATGACACCGCCGCTGGAGCACGACTACATAGGCCTCTCGGAGAGCCCTGACAAGTTCTTGGCTGCCGGGGCGCTCAATCTCAAGGACACTGAGCTCCGGCTTGGACTCCCGGGTTCGGAGTCGCCGGAGAGGGCGGATGGAGCAGGGGTCGGGCTGTGGCTGGGGCCGCCCAAGGGCTTCGTCTCCGGGGCCAAGAGGGGGTTTTCGGACACTATCGAAGAGGCCGGAAAGTGGGGGCTTCCTGGAGTGAATGGAACTGAACTGGAGCCTGGGAAGGGTGGGGTTTCCTTATCCGCCAAGGCGGAGAACGCCGGTGGAAAGCAATCAGGACCGGCCAGCGCAGGGAAGGGTGCCGGTGGTGTCGAAAAGACAGCGCCTTCGCCAAAGGCACAAGTGGTTGGATGGCCACCGATCCGAAGTTATAGAAAGAACACTATGGTCGCAAACCCAACCAAGAAAAAGGAGGATGCTGAAGTGAAACAAGGGGTGGAATGCCTCTATATCAAGGTCAGCATGGATGGAGCTCCATATCTCAGAAAAGTTGACCTCAAAACGTATTCCGACTATAAGGAACTCTCACTGGCTCTTGAGAAGATGTTTACTTGCTTTACTATTGGTCAGTGTGGATCACATGAAATGACCAACAGAGAGATGTTAACAGAGGGTCGAGTGATAGATCTCCTTGAAGGGTCTGAATATGTTCTTACCTATGAGGACAAGGATGGAGATTGGATGCTTGTGGGCGATGTTCCATGGAGCATGTTTACTGAATCATGCAGAAGGTTGAGGATCATGAAAGGTTCAGATGCAATTGGTATCGCTCCAAGGGCCATGGAGAAATCCAAGAGCAGGAACTAA

**Protein sequence**

MTPPLEHDYIGLSESPDKFLAAGALNLKDTELRLGLPGSESPERADGAGVGLWLGPPKGFVSGAKRGFSDTIEEAGKWGLPGVNGTELEPGKGGVSLSAKAENAGGKQSGPASAGKGAGGVEKTAPSPKAQVVGWPPIRSYRKNTMVANPTKKKEDAEVKQGVECLYIKVSMDGAPYLRKVDLKTYSDYKELSLALEKMFTCFTIGQCGSHEMTNREMLTEGRVIDLLEGSEYVLTYEDKDGDWMLVGDVPWSMFTESCRRLRIMKGSDAIGIAPRAMEKSKSRN

**HcIAA22 >evm.model.scaf_42.52**

ATGTCGACGACGGAGACGGCGCACAGCTCCACTGAATCTGATGCCTCCGGCCTAGACTACGAAGAGACGCAGCTCACGCTCGCCCCGCCCGGTGCCCCCAGATCCGACCCTGAAAGGAAGCGAGGCCTATCGGAGGACGATCTCCCCTCCGATTCCTCCCAAGGACCCTCCCGAAAACCTACACAATCCAAGGCTCAAGTGGTGGGATGGCCGCCGGTGAGGTCGTTCCGGCGAAACGCGCTCAAGAGCTGCACCTACGTGAAGGTGGCCGTCGACGGTGCGCCGTACCTTCGTAAGGTTGACCTGGAGGCCTATGCCGGATACCAGCAGTTGCTTACTGCACTCGAGGAAATGTTCTCTTGCTTCACTGCCCGCAATTATCCCAATGAGAGGAGGCTGGTTGATCCTGCGAGTGGCGCAGAGTATGTGCCGACTTATGAGGACAAAGATGGTGATTGGATGCTTGTCGGTGATGTTCCCTGGAAAATGTTCGTTGCTTCATGCAAGCGTCTTAGGTTAATGAAAATCTCTGAAGCTATCAATTTAGCTCCAAGAGCACCACAAGGATGCACCAAAGAACAGTGA

**Protein sequence**

MSTTETAHSSTESDASGLDYEETQLTLAPPGAPRSDPERKRGLSEDDLPSDSSQGPSRKPTQSKAQVVGWPPVRSFRRNALKSCTYVKVAVDGAPYLRKVDLEAYAGYQQLLTALEEMFSCFTARNYPNERRLVDPASGAEYVPTYEDKDGDWMLVGDVPWKMFVASCKRLRLMKISEAINLAPRAPQGCTKEQ

**HcIAA23 >evm.model.scaf_270.58**

ATGGCATCATATAAAATAGTGGGAATTGAAGGAGCAAAATGTAAAAAAAAACAAAATCAATTTACTGCCCTTCATGACTATAAATTGAGCATCCTTTCTCTTCTTCTCCTCCTCCCAAACTCCTCTCTTCCTATCATCACCAATACAAGGAAATGGAAGCAGAGATGGACAACCTCAAGGCCACCTGAGCTTAGGCTCGGCCTTCCGGGTTGTGGTGCCTCTGACGATGCACAGCCAGTGCCCTCGAGGCTGTCTAAGCGCTCCCTGGACGACCACACCCATGCCCGGAAAACACAGCTAGTTGGGTGGCCGCCAGTTCGCTCTTACCGGAAGAACGCAATGAAGACTGAAGCAGCCGGTTTTTTTGTCAAGGTCAGCATGGATGGCGCCCCTTACTTAAGGAAGATCGATCTGAGGGTGTACAAGGGCTACAAGGAACTAAGGGAGGGGCTGGAGGACATGTTCAAGGTCTTCGAGGGCAGCAAAGGGTCGGAATATGCCATCACCTACGAAGACAAAGATGGGGATTTGATGCTCGTTGGAGATGTTCCATGGGAGATGTTCACATCTTCTTGCAAGAAGCTGAGGATTATAAGAGGGTCTGAGTCAACTTGA

**Protein sequence**

MASYKIVGIEGAKCKKKQNQFTALHDYKLSILSLLLLLPNSSLPIITNTRKWKQRWTTSRPPELRLGLPGCGASDDAQPVPSRLSKRSLDDHTHARKTQLVGWPPVRSYRKNAMKTEAAGFFVKVSMDGAPYLRKIDLRVYKGYKELREGLEDMFKVFEGSKGSEYAITYEDKDGDLMLVGDVPWEMFTSSCKKLRIIRGSEST

**HcIAA24 >evm.model.scaf_484.51.1**

ATGGAAGGAGAAGACTTCAAGAACGGGGGACATGGATGTCCTCGGCTGCTAGATTTGATCCCAAATGTGAGAGTGGTGCAAGATGGAGGAGGGGGAAGAGGAAGAAGAAGAACGTTTGGGCGTTTAGATGCCTCAGAAGAAATGAAACTGGAACTGAGGCTTGGACTCCCTGGAGGAGACTTAGAAGAAGAAGAAGAGTCCTCTGTTTTATCGCTTGGTTTTATCTCCAAGGCTTCAAAAACCTCCTCCGTTAGGGTCTTTGGAGCAGTTAAATCTAAAAATGAAGGTTTTCGGCATCAGGAAACTGGATTTTTCAAGTTGCAGAGCCGAGCTGGTACTGAAAAGGAACACAATACCACTTGGGCTAATGGGGAATTTGAACAACAACAAAACCTCGAGAAGGAGAAAGCAGATGGAACTGCTGGACCCAGCACCAGCTCCCAGACGAGAGCTGCTGCTGTTCCTGTTGTTGGTTGGCCTCCCATCAGATCCTTCAGAAAGAATCTAGCAACTAATTCAGCCAAACAAACAGTGGAGCCAGAGACTAGAAGAATAGTGGAACAAGTGAAACTTGAGAACAACAAGGGTTTGCTCGTGAAGATCAACATGGATGGTATCCCAATTGGGAGAAAAGTTGATTTAAAAGCTTATGATAGCTATGAGAAGCTCTCGAATGCAGTGGAAGATCTCTTTCTAGGCCTTTTTGAAGCTCAAAAGGATATCTCTGCAAATGCCATTCAGAAGGATGAGGAAGCAAAAAGAGCATTCACAGGTTTAATGGATGACTCTGGTGACTACACTTTGGTTTATGAAGACAATCATGGTCATAGAATGCTTGTTGGGGATGTTCCATGGGACACTTTCAGCTCGACTACTGCTTGTTCCCTCTTAGAAAGTCATCGTAGATGCATGCCTATGGCCAGCAAGGATGTTTGTTTCAACTGTAAAAAGGTTGAGGGTGTTGAAAAGTTCTCATCTTTCTTCATTACGTTTAGGATCTGTATGCCGGAAAAGAACAGTGACAGAGTGTTGAGATCGGTGGAAGGCCTTTGGGACTGA

**Protein sequence**

MEGEDFKNGGHGCPRLLDLIPNVRVVQDGGGGRGRRRTFGRLDASEEMKLELRLGLPGGDLEEEEESSVLSLGFISKASKTSSVRVFGAVKSKNEGFRHQETGFFKLQSRAGTEKEHNTTWANGEFEQQQNLEKEKADGTAGPSTSSQTRAAAVPVVGWPPIRSFRKNLATNSAKQTVEPETRRIVEQVKLENNKGLLVKINMDGIPIGRKVDLKAYDSYEKLSNAVEDLFLGLFEAQKDISANAIQKDEEAKRAFTGLMDDSGDYTLVYEDNHGHRMLVGDVPWDTFSSTTACSLLESHRRCMPMASKDVCFNCKKVEGVEKFSSFFITFRICMPEKNSDRVLRSVEGLWD

**HcIAA25 >evm.model.scaf_566.42**

ATGGCGAGCTACGTCGAGGATACCGAGCTACGCCTCGGTTTGCCGGGATCCGATGCGCCGGCTACAAGGGGTAGCAAAAGGTCGCTGCCGGAGGATGACGCTGCAGGCGACGACTCCAGCTGCCGAAATGGCGGATCTGGCTCGGCGGCCAAGGCTCAAGTGGTTGGATGGCCGCCGATCCGAACGTACAGGAAGAACAGCTTCCAGGCGATGAAGGTAGAGGCGAAGGTGGAGCCGGAGGCTTCTGGGTTGTTCGTGAAGGTTAGCATGGATGGAGCTCCTTACTTGAGGAAGATTGATCTCAAGGTTTACAAGGGGTACAAGGAGCTCAGGGACGGCTTGGACGGCATGTTCAACTGCTTCTCTCAAGTAGAATTGACAAGAAAAGAAGGGTGCAATGGATCTGAATATGCCATAACTTACGAAGACAAAGATGGAGATTTGATGCTGGTCGGAGATGTTCCTTGGGAGATGTTTATATGTTCTTGCAAAAGGCTAAGGATAATGAAAGGATCTGAAGCAAGAGGTTTGGAATCCAGACAATAA

**Protein sequence**

MASYVEDTELRLGLPGSDAPATRGSKRSLPEDDAAGDDSSCRNGGSGSAAKAQVVGWPPIRTYRKNSFQAMKVEAKVEPEASGLFVKVSMDGAPYLRKIDLKVYKGYKELRDGLDGMFNCFSQVELTRKEGCNGSEYAITYEDKDGDLMLVGDVPWEMFICSCKRLRIMKGSEARGLESRQ

**HcIAA26 >evm.model.scaf_641.57**

ATGGCGAGTGAATTGGGGTTGGAGGAGACGGAGCTGCGTCTGGGACTGGGCTTGCCCTGCGGGGGCGGCGGCGGAGGGGAAGGCGAGGGCGCGAAGAAGAGGGGATTCGAGGAGACCATTGATCTGAAGCTTCAGCTTCAGACTCCGACGGAGGCGAAGGAATCGGCGGCGGCCATGCAATCTGCCGACAATATGACGAAGAAGACGCCGAGCCTTGTGAACATTGCGTCTTGTGGCGGCTGCGTCGACCCGGAAAAGCCGCCTGCTGCCAAGGCCCAGGTTGTGGGTTGGCCACCAGTGAGATCCTTCAGGAAGAACATCCTCTCTGTTCACTCTGAGAAGCGTGCCGGCAACAGCAAGGATGACAGCGAAAAGTTCGGCAACTCTCCGGCCGCCTTTGTGAAGGTGAGCATGGATGGCGCGCCATACCTGCGCAAGGTGGACCTAAAGATGTACAAGAGCTACCAAGAGCTTTCCATGTCCTTGCAGAAGATGTTTAGCTCCTTCACCACCAGCACTGAAAACAGTGGCTCTCAGGGAATGAGTGGGAGGGACTACATGAATGAGAGCAAGGTGATGGATCTGCTGAATGGATCTGAGTATGTGCCAACCTATGAAGACAAGGATGGAGACTGGATGCTTGTCGGTGATGTTCCATGGGAGATGTTTGTCGACTCGTGCAAACGTTTGCGTATCATGAAGGGATCAGAAGCCATTGGAATTGCACCAAGAGCCATGGAGAAGCGCAAGAACAGAAGCTGA

**Protein sequence**

MASELGLEETELRLGLGLPCGGGGGGEGEGAKKRGFEETIDLKLQLQTPTEAKESAAAMQSADNMTKKTPSLVNIASCGGCVDPEKPPAAKAQVVGWPPVRSFRKNILSVHSEKRAGNSKDDSEKFGNSPAAFVKVSMDGAPYLRKVDLKMYKSYQELSMSLQKMFSSFTTSTENSGSQGMSGRDYMNESKVMDLLNGSEYVPTYEDKDGDWMLVGDVPWEMFVDSCKRLRIMKGSEAIGIAPRAMEKRKNRS

**HcIAA27 >evm.model.scaf_326.19**

ATGGGAGGATACTGTAACTCCCCTTCTTCTTCCATAGACAGCAGCTGTGACCCTGACCTCAGCACATCTTCCATCTTTGAGCAAAGGAGAGACTTGAGCACTGAGCTAAGGCTAGGCCTTAGTCTCTCTTCATCCTCTTTACAACACAACTCTCATAAGGAAAAGGATCCACTATCTGACTGGCCACCAATCAAACCACTTCTTAGGAGCACACTCGAGGGCAAAACGCATCAGAGTAACTCGGAGACATTCTTTGTGAAGGTTTACATGGAGGGAGTTCCAATTGGCAGGAAACTCGACTTGTTCGCGCATAATGGCTACGGTGATCTGATAAAGTCCCTTCGTCGCATGTTTAGGACGACGATCACATGCTCTCATGTTTCCCAAGTGCCGCTAGAGAAGAGCCATGTGCTCACTTATGAGGACGAAGAGGGAGATTGGATGATGGTCGGAGATGTGCCATGGGAGTTGTTCTTGAGCACCGTCAAGAAGTTGAAGATCACTAGAGTTGACAAATGCTAG

**Protein sequence**

MGGYCNSPSSSIDSSCDPDLSTSSIFEQRRDLSTELRLGLSLSSSSLQHNSHKEKDPLSDWPPIKPLLRSTLEGKTHQSNSETFFVKVYMEGVPIGRKLDLFAHNGYGDLIKSLRRMFRTTITCSHVSQVPLEKSHVLTYEDEEGDWMMVGDVPWELFLSTVKKLKITRVDKC
