# Supplementary figures and images for: Genome-Wide Analysis and Characterization of the Aux/IAA Family Genes Related to Floral Scent Formation in Hedychium coronarium
Source: Int J Mol Sci. 2019 Jul 1;20(13):3235. doi: 10.3390/ijms20133235 (PMC6651449; doi:10.3390/ijms20133235)

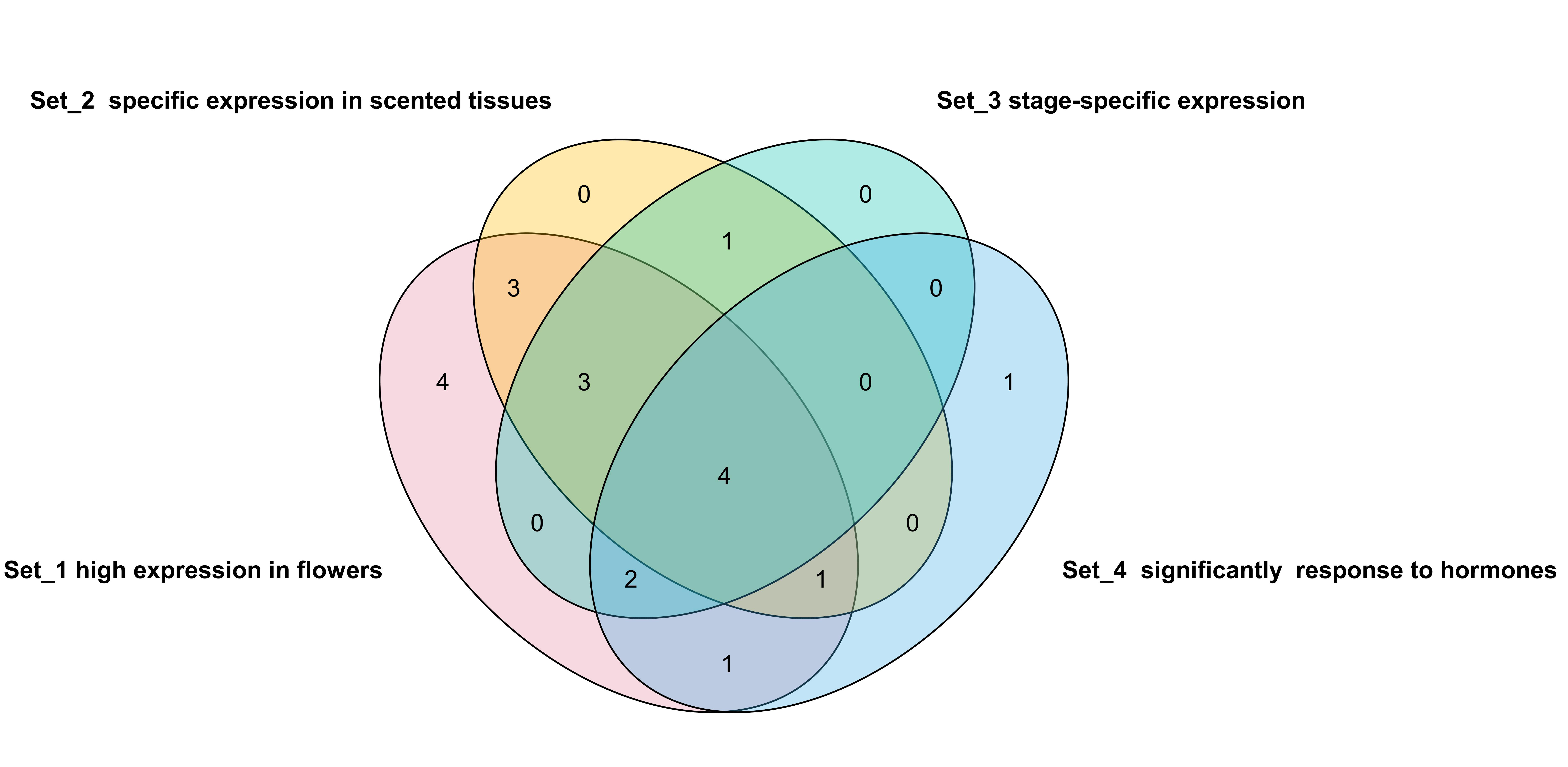

Supplement: Supplementary file 1 [file ijms-20-03235-s001.zip › ijms-514365 supp final/ijms-514365 supp/Supplementary Figure S1.tif]
