# Supplementary material for: Genome-Wide Analysis and Characterization of the Aux/IAA Family Genes Related to Floral Scent Formation in Hedychium coronarium
Source: Int J Mol Sci. 2019 Jul 1;20(13):3235. doi: 10.3390/ijms20133235 (PMC6651449; doi:10.3390/ijms20133235)
Supplement: Supplementary file 1 [file ijms-20-03235-s001.zip › ijms-514365 supp final/ijms-514365 supp/Supplementary Table S2.doc]

|  | HcIAA1 | HcIAA2 | HcIAA3 | HcIAA4 | HcIAA5 | HcIAA6 | HcIAA7 | HcIAA8 | HcIAA9 | HcIAA10 | HcIAA11 | HcIAA12 | HcIAA13 | HcIAA14 | HcIAA15 | HcIAA16 | HcIAA17 | HcIAA18 | HcIAA19 | HcIAA20 | HcIAA21 | HcIAA22 | HcIAA23 | HcIAA24 | HcIAA25 | HcIAA26 | HcIAA27 |
| --- | --- | --- | --- | --- | --- | --- | --- | --- | --- | --- | --- | --- | --- | --- | --- | --- | --- | --- | --- | --- | --- | --- | --- | --- | --- | --- | --- |
| HcIAA1 | 1.00 | 0.44 | 0.17 | 0.24 | 0.23 | 0.39 | 0.38 | 0.44 | 0.46 | 0.44 | 0.25 | 0.40 | 0.22 | 0.44 | 0.39 | 0.46 | 0.25 | 0.21 | 0.20 | 0.40 | 0.45 | 0.41 | 0.23 | 0.16 | 0.42 | 0.43 | 0.23 |
| HcIAA2 | 0.44 | 1.00 | 0.24 | 0.27 | 0.28 | 0.42 | 0.31 | 0.41 | 0.44 | 0.40 | 0.27 | 0.33 | 0.27 | 0.61 | 0.79 | 0.40 | 0.27 | 0.25 | 0.26 | 0.37 | 0.45 | 0.39 | 0.30 | 0.20 | 0.41 | 0.64 | 0.21 |
| HcIAA3 | 0.17 | 0.24 | 1.00 | 0.21 | 0.24 | 0.27 | 0.29 | 0.22 | 0.21 | 0.20 | 0.22 | 0.17 | 0.21 | 0.25 | 0.25 | 0.26 | 0.77 | 0.19 | 0.19 | 0.20 | 0.21 | 0.26 | 0.28 | 0.16 | 0.25 | 0.25 | 0.31 |
| HcIAA4 | 0.24 | 0.27 | 0.21 | 1.00 | 0.27 | 0.26 | 0.23 | 0.29 | 0.30 | 0.28 | 0.40 | 0.23 | 0.55 | 0.28 | 0.28 | 0.26 | 0.22 | 0.42 | 0.27 | 0.25 | 0.28 | 0.23 | 0.22 | 0.35 | 0.26 | 0.31 | 0.20 |
| HcIAA5 | 0.23 | 0.28 | 0.24 | 0.27 | 1.00 | 0.25 | 0.19 | 0.28 | 0.27 | 0.26 | 0.25 | 0.21 | 0.26 | 0.27 | 0.28 | 0.22 | 0.22 | 0.28 | 0.49 | 0.25 | 0.28 | 0.26 | 0.24 | 0.21 | 0.26 | 0.27 | 0.20 |
| HcIAA6 | 0.39 | 0.42 | 0.27 | 0.26 | 0.25 | 1.00 | 0.49 | 0.39 | 0.36 | 0.34 | 0.28 | 0.31 | 0.26 | 0.42 | 0.41 | 0.58 | 0.28 | 0.25 | 0.22 | 0.35 | 0.37 | 0.40 | 0.43 | 0.21 | 0.60 | 0.41 | 0.23 |
| HcIAA7 | 0.38 | 0.31 | 0.29 | 0.23 | 0.19 | 0.49 | 1.00 | 0.33 | 0.32 | 0.30 | 0.24 | 0.29 | 0.22 | 0.33 | 0.29 | 0.52 | 0.24 | 0.18 | 0.21 | 0.28 | 0.32 | 0.39 | 0.55 | 0.17 | 0.53 | 0.33 | 0.28 |
| HcIAA8 | 0.44 | 0.41 | 0.22 | 0.29 | 0.28 | 0.39 | 0.33 | 1.00 | 0.62 | 0.60 | 0.29 | 0.40 | 0.26 | 0.41 | 0.39 | 0.38 | 0.22 | 0.27 | 0.26 | 0.55 | 0.59 | 0.34 | 0.34 | 0.27 | 0.37 | 0.42 | 0.18 |
| HcIAA9 | 0.46 | 0.44 | 0.21 | 0.30 | 0.27 | 0.36 | 0.32 | 0.62 | 1.00 | 0.87 | 0.27 | 0.40 | 0.28 | 0.43 | 0.40 | 0.35 | 0.22 | 0.28 | 0.26 | 0.72 | 0.63 | 0.33 | 0.31 | 0.23 | 0.35 | 0.43 | 0.17 |
| HcIAA10 | 0.44 | 0.40 | 0.20 | 0.28 | 0.26 | 0.34 | 0.30 | 0.60 | 0.87 | 1.00 | 0.27 | 0.37 | 0.29 | 0.42 | 0.38 | 0.33 | 0.21 | 0.27 | 0.26 | 0.72 | 0.63 | 0.32 | 0.30 | 0.23 | 0.33 | 0.43 | 0.16 |
| HcIAA11 | 0.25 | 0.27 | 0.22 | 0.40 | 0.25 | 0.28 | 0.24 | 0.29 | 0.27 | 0.27 | 1.00 | 0.23 | 0.37 | 0.30 | 0.29 | 0.29 | 0.23 | 0.39 | 0.24 | 0.26 | 0.30 | 0.24 | 0.23 | 0.34 | 0.26 | 0.28 | 0.17 |
| HcIAA12 | 0.40 | 0.33 | 0.17 | 0.23 | 0.21 | 0.31 | 0.29 | 0.40 | 0.40 | 0.37 | 0.23 | 1.00 | 0.19 | 0.35 | 0.30 | 0.29 | 0.21 | 0.22 | 0.19 | 0.36 | 0.46 | 0.29 | 0.21 | 0.18 | 0.31 | 0.37 | 0.19 |
| HcIAA13 | 0.22 | 0.27 | 0.21 | 0.55 | 0.26 | 0.26 | 0.22 | 0.26 | 0.28 | 0.29 | 0.37 | 0.19 | 1.00 | 0.28 | 0.29 | 0.26 | 0.22 | 0.38 | 0.26 | 0.28 | 0.27 | 0.24 | 0.20 | 0.30 | 0.23 | 0.29 | 0.19 |
| HcIAA14 | 0.44 | 0.61 | 0.25 | 0.28 | 0.27 | 0.42 | 0.33 | 0.41 | 0.43 | 0.42 | 0.30 | 0.35 | 0.28 | 1.00 | 0.59 | 0.40 | 0.26 | 0.26 | 0.28 | 0.37 | 0.44 | 0.42 | 0.33 | 0.20 | 0.43 | 0.76 | 0.21 |
| HcIAA15 | 0.39 | 0.79 | 0.25 | 0.28 | 0.28 | 0.41 | 0.29 | 0.39 | 0.40 | 0.38 | 0.29 | 0.30 | 0.29 | 0.59 | 1.00 | 0.41 | 0.28 | 0.26 | 0.19 | 0.35 | 0.40 | 0.40 | 0.31 | 0.22 | 0.43 | 0.60 | 0.21 |
| HcIAA16 | 0.46 | 0.40 | 0.26 | 0.26 | 0.22 | 0.58 | 0.52 | 0.38 | 0.35 | 0.33 | 0.29 | 0.29 | 0.26 | 0.40 | 0.41 | 1.00 | 0.23 | 0.22 | 0.28 | 0.31 | 0.36 | 0.40 | 0.42 | 0.18 | 0.82 | 0.40 | 0.27 |
| HcIAA17 | 0.25 | 0.27 | 0.77 | 0.22 | 0.22 | 0.28 | 0.24 | 0.22 | 0.22 | 0.21 | 0.23 | 0.21 | 0.22 | 0.26 | 0.28 | 0.23 | 1.00 | 0.20 | 0.20 | 0.21 | 0.21 | 0.25 | 0.22 | 0.17 | 0.24 | 0.27 | 0.26 |
| HcIAA18 | 0.21 | 0.25 | 0.19 | 0.42 | 0.28 | 0.25 | 0.18 | 0.27 | 0.28 | 0.27 | 0.39 | 0.22 | 0.38 | 0.26 | 0.26 | 0.22 | 0.20 | 1.00 | 0.25 | 0.27 | 0.29 | 0.20 | 0.20 | 0.47 | 0.25 | 0.26 | 0.15 |
| HcIAA19 | 0.20 | 0.26 | 0.19 | 0.27 | 0.49 | 0.22 | 0.21 | 0.26 | 0.26 | 0.26 | 0.24 | 0.19 | 0.26 | 0.28 | 0.19 | 0.28 | 0.20 | 0.25 | 1.00 | 0.25 | 0.28 | 0.24 | 0.21 | 0.22 | 0.21 | 0.27 | 0.20 |
| HcIAA20 | 0.40 | 0.37 | 0.20 | 0.25 | 0.25 | 0.35 | 0.28 | 0.55 | 0.72 | 0.72 | 0.26 | 0.36 | 0.28 | 0.37 | 0.35 | 0.31 | 0.21 | 0.27 | 0.25 | 1.00 | 0.57 | 0.32 | 0.26 | 0.23 | 0.29 | 0.37 | 0.16 |
| HcIAA21 | 0.45 | 0.45 | 0.21 | 0.28 | 0.28 | 0.37 | 0.32 | 0.59 | 0.63 | 0.63 | 0.30 | 0.46 | 0.27 | 0.44 | 0.40 | 0.36 | 0.21 | 0.29 | 0.28 | 0.57 | 1.00 | 0.36 | 0.31 | 0.24 | 0.37 | 0.44 | 0.17 |
| HcIAA22 | 0.41 | 0.39 | 0.26 | 0.23 | 0.26 | 0.40 | 0.39 | 0.34 | 0.33 | 0.32 | 0.24 | 0.29 | 0.24 | 0.42 | 0.40 | 0.40 | 0.25 | 0.20 | 0.24 | 0.32 | 0.36 | 1.00 | 0.33 | 0.16 | 0.41 | 0.40 | 0.22 |
| HcIAA23 | 0.23 | 0.30 | 0.28 | 0.22 | 0.24 | 0.43 | 0.55 | 0.34 | 0.31 | 0.30 | 0.23 | 0.21 | 0.20 | 0.33 | 0.31 | 0.42 | 0.22 | 0.20 | 0.21 | 0.26 | 0.31 | 0.33 | 1.00 | 0.18 | 0.45 | 0.32 | 0.29 |
| HcIAA24 | 0.16 | 0.20 | 0.16 | 0.35 | 0.21 | 0.21 | 0.17 | 0.27 | 0.23 | 0.23 | 0.34 | 0.18 | 0.30 | 0.20 | 0.22 | 0.18 | 0.17 | 0.47 | 0.22 | 0.23 | 0.24 | 0.16 | 0.18 | 1.00 | 0.18 | 0.20 | 0.14 |
| HcIAA25 | 0.42 | 0.41 | 0.25 | 0.26 | 0.26 | 0.60 | 0.53 | 0.37 | 0.35 | 0.33 | 0.26 | 0.31 | 0.23 | 0.43 | 0.43 | 0.82 | 0.24 | 0.25 | 0.21 | 0.29 | 0.37 | 0.41 | 0.45 | 0.18 | 1.00 | 0.41 | 0.27 |
| HcIAA26 | 0.43 | 0.64 | 0.25 | 0.31 | 0.27 | 0.41 | 0.33 | 0.42 | 0.43 | 0.43 | 0.28 | 0.37 | 0.29 | 0.76 | 0.60 | 0.40 | 0.27 | 0.26 | 0.27 | 0.37 | 0.44 | 0.40 | 0.32 | 0.20 | 0.41 | 1.00 | 0.21 |
| HcIAA27 | 0.23 | 0.21 | 0.31 | 0.20 | 0.20 | 0.23 | 0.28 | 0.18 | 0.17 | 0.16 | 0.17 | 0.19 | 0.19 | 0.21 | 0.21 | 0.27 | 0.26 | 0.15 | 0.20 | 0.16 | 0.17 | 0.22 | 0.29 | 0.14 | 0.27 | 0.21 | 1.00 |

Table S2. The similarity among Aux/IAA proteins in *H. coronarium*
